# Fine-Scale Map Reveals Highly Variable Recombination Rates Associated with Genomic Features in the Eurasian Blackcap

**DOI:** 10.1093/gbe/evad233

**Published:** 2024-01-10

**Authors:** Karen Bascón-Cardozo, Andrea Bours, Georg Manthey, Gillian Durieux, Julien Y Dutheil, Peter Pruisscher, Linda Odenthal-Hesse, Miriam Liedvogel

**Affiliations:** MPRG Behavioural Genomics, Max Planck Institute for Evolutionary Biology, Plön 24306, Germany; MPRG Behavioural Genomics, Max Planck Institute for Evolutionary Biology, Plön 24306, Germany; Institute of Avian Research “Vogelwarte Helgoland”, Wilhelmshaven 26386, Germany; MPRG Behavioural Genomics, Max Planck Institute for Evolutionary Biology, Plön 24306, Germany; Department for Theoretical Biology, Max Planck Institute for Evolutionary Biology, Plön 24306, Germany; MPRG Behavioural Genomics, Max Planck Institute for Evolutionary Biology, Plön 24306, Germany; Department of Zoology, Stockholm University, Stockholm SE-106 91, Sweden; Department Evolutionary Genetics, Max Planck Institute for Evolutionary Biology, Plön 24306, Germany; MPRG Behavioural Genomics, Max Planck Institute for Evolutionary Biology, Plön 24306, Germany; Institute of Avian Research “Vogelwarte Helgoland”, Wilhelmshaven 26386, Germany; Department of Biology and Environmental Sciences, Carl von Ossietzky University of Oldenburg, Oldenburg 26129, Germany

**Keywords:** historical recombination rates, bird genome, transposable elements, CpG islands, regulatory elements

## Abstract

Recombination is responsible for breaking up haplotypes, influencing genetic variability, and the efficacy of selection. Bird genomes lack the protein PR domain-containing protein 9, a key determinant of recombination dynamics in most metazoans. Historical recombination maps in birds show an apparent stasis in positioning recombination events. This highly conserved recombination pattern over long timescales may constrain the evolution of recombination in birds. At the same time, extensive variation in recombination rate is observed across the genome and between different species of birds. Here, we characterize the fine-scale historical recombination map of an iconic migratory songbird, the Eurasian blackcap (*Sylvia atricapilla*), using a linkage disequilibrium–based approach that accounts for population demography. Our results reveal variable recombination rates among and within chromosomes, which associate positively with nucleotide diversity and GC content and negatively with chromosome size. Recombination rates increased significantly at regulatory regions but not necessarily at gene bodies. CpG islands are associated strongly with recombination rates, though their specific position and local DNA methylation patterns likely influence this relationship. The association with retrotransposons varied according to specific family and location. Our results also provide evidence of heterogeneous intrachromosomal conservation of recombination maps between the blackcap and its closest sister taxon, the garden warbler. These findings highlight the considerable variability of recombination rates at different scales and the role of specific genomic features in shaping this variation. This study opens the possibility of further investigating the impact of recombination on specific population-genomic features.

**Significance**

The exchange of genetic information between parental chromosomes generates new combinations of alleles in the offspring during meiotic recombination. Recombination events are not spread randomly across the genome and can vary widely at different scales. We used a bioinformatic tool, which considers the fluctuation of population sizes through many generations, to estimate historical recombination rates in the iconic blackcap. Our results show that the variation of recombination frequency is associated with specific genomic features and recombination events are prevalent in regulatory regions. Recombination patterns were differentially conserved between closely related species. This study highlights the importance of understanding the genomic architecture and fine-scale recombination landscape in species focally studied in an evolutionary context.

## Introduction

Meiotic recombination reshuffles parental genetic material, thus creating new combinations of alleles, providing the primary source of genetic and haplotype diversity. The immediate benefit of recombination includes increased reproductive success and fitness of offspring by promoting proper chromosome disjunction and avoiding aneuploidy (Hassold and Hunt 2001, Kong et al. 2004). Indirect advantage results when recombination impacts selection efficacy at linked loci under Hill–Roberson interference (Hill and Robertson 1966; Betancourt et al. 2009; Hickey and Golding 2018). Breaking up nonrandom associations between loci across the genome increases the probability of advantageous alleles fixing and impedes deleterious alleles’ propagation. Hence, recombination is a critical player influencing the rate of genetic diversity, introgression, differentiation, and, subsequently, speciation (Butlin 2005; Nachman and Payseur 2012; Mugal et al. 2013; Martin et al. 2019). Additionally, recombination is associated with genome evolution and may contribute to maintaining genes important for specific behavioral traits (Kent et al. 2012).

In many vertebrates, the positioning of recombination events is determined in *trans* by the meiotic methyltransferase PR domain-containing protein 9 (PRDM9), which binds specific nucleotide motifs via a variable zinc finger DNA-binding domain (Baudat et al. 2010; Parvanov et al. 2010; Berg et al. 2011). Genome-wide location and frequency of recombination events, often called recombination maps, change rapidly due to a combination of extensive evolutionary turnover of the zinc fingers that confer specificity to DNA motifs and subsequent erosion of the motifs via biased gene conversion (Baudat et al. 2010; Myers et al. 2010; Parvanov et al. 2010). PRDM9 function across the tree of life is not fully understood, but in organisms that possess functional PRDM9, like mice and humans, recombination events cluster in intergenic regions away from functional elements (Brick et al. 2012; Pratto et al. 2014).

However, PRDM9 has been lost multiple times in the metazoan tree of life, including mammals such as platypus and dogs, several clades of ray-finned fish, amphibians, some reptiles, lizards, as well as in the lineage leading to crocodiles and birds (Baker et al. 2017; Cavassim et al. 2022). For some of these PRDM9-deficient species, contemporary recombination occurs at the locations of existing open chromatin marks, such as promoters or functional regions where access to the DNA for the transcription machinery is enhanced. Such placement in the default open chromatin has been described for plants (Choi et al. 2013; Apuli et al. 2020), yeast (Lam and Keeney 2015), some species of fish (Shanfelter et al. 2019), canids (Auton et al. 2013), and birds (Singhal et al. 2015; Kawakami et al. 2017).

Birds lack PRDM9 and possess compact genomes, containing a high density of coding regions, especially in microchromosomes, and only a few repetitive elements (Zhang et al. 2014). This facilitates the generation of high-quality reference genomes at the chromosome scale, enabling the generation of fine-scale genetic linkage maps from which contemporary recombination rates can be inferred. Earlier linkage maps based on few genetic markers corroborated the inverse relationship between chromosome size and recombination rate (Groenen et al. 2000; Stapley et al. 2008), as well as interspecies differences in the degree of heterochiasmy, i.e. the difference in recombination rate between sexes (Hansson et al. 2005; Akesson et al. 2007; Backström, Karaiskou, et al. 2008). Recombination estimation with linkage-based approaches depends on the number of variable sites (single-nucleotide polymorphic sites [SNPs]) that can be analyzed. Lower marker densities typically report lower recombination rates (see supplementary table S1, Supplementary Material online). Nevertheless, even in high-resolution analyses based on whole-genome resequencing (WGS), recombination rates vary with genome-wide rate of 1.5 cM/Mb in fairy-wrens (Peñalba et al. 2020), 1.8 cM/Mb in honeyeaters (Robledo-Ruiz et al. 2022), and 2 cM/Mb in the house sparrow, with higher recombination rates for microchromosomes (6.41 cM/Mb) compared with macrochromosomes (1.78 cM/Mb) (Hagen et al. 2020). Thus, genome-wide recombination rates differ not only along the genome but also between different (sub)species of birds (Backström et al. 2010; Kawakami et al. 2014; Peñalba et al. 2020).

Mechanistically, recombination events occur during Meiosis-I, when specific chromosomal regions receive programmed double-strand breaks (DSBs), which are then repaired via the exchange of genetic material as nonreciprocal gene conversion events or reciprocal crossing-over (CO). At least 1 obligate CO occurs on macrobivalents to prevent nondisjunction. Precise estimates of the rate and distribution of recombination events can be directly observed as chiasma (chromosomal strand exchanges) in germ cells or by immunofluorescence analysis of recombination proteins such as Mut-l-homolog 1 (MLH1) protein foci that mark the sites of successful CO recombination in recombination nodules. Across most bird species, karyotype and synteny are highly conserved (Kayang et al. 2006; Backström, Fagerberg, et al. 2008; Stapley et al. 2008; Ellegren 2010). Recombination rates are high, with the average number of total MLH1 foci in all chromosomes being 57.4 ± 8.1 across all birds with available cytological data (supplementary table S1, Supplementary Material online). Nevertheless, while the average number of recombination nodules on the largest macrobivalents (the largest homologous chromosome pair) is 6.1 ± 2.6 in oocytes and 5.7 ± 1.8 in spermatocytes across various tested bird species (see supplementary table S1, Supplementary Material online), only 2 foci are found on the largest macrobivalent in the zebra finch, with oocytes having 2.1 ± 0.4 and spermatocytes possessing 2.3 ± 0.5 foci. Intermediate numbers seen in common swift (3.8 ± 1.1) and Eurasian hobby (4.8 ± 1.1) (Malinovskaya et al. 2018), with as many as 9.0 ± 1.4 foci, are seen in chicken oocytes (Pigozzi 2001). Therefore, while variability in recombination rate occurs within a narrow limit (Malinovskaya et al. 2018), interchromosomal variation in recombination rate and patterning is common.

In summary, contemporary recombination rates determined using genome linkage and cytological data reveal significant differences between and within the same species. From an evolutionary and ecological perspective, it is crucial to understand variation in recombination rates and the causes and consequences of this variability. However, cytological and linkage maps can only provide insight into contemporary recombination rates, while population-based recombination rates (historical recombination maps) based on linkage disequilibrium (LD) analyses are the only tool that allows the assessment of the evolution of recombination across time.

Historical recombination maps, inferred from an accumulation of CO recombination events over long evolutionary timescales, reflect the genome-wide placement of recombination events, so-called recombination landscapes. Historical LD-based studies in passerines are rare. The only currently available studies on historical recombination rates in birds stem from zebra finches and flycatchers (Singhal et al. 2015; Kawakami et al. 2017). Notably, population size fluctuations (Kamm et al. 2016; Dapper and Payseur 2018) and gene flow (Samuk and Noor 2022) have been shown to heavily influence the accuracy of historical recombination inference at finer scales, and these studies did not consider underlying population demography. While the historical recombination rate was comparable with the contemporary recombination rate in the collared flycatcher (Kawakami et al. 2017), LD-based inferences without demography revealed a much lower historical recombination rate compared with contemporary rates determined by linkage maps and cytological data of the zebra finch (Singhal et al. 2015).

Interestingly, a significant overlap of historical recombination landscapes across closely related species was reported to persist over long evolutionary timescales (Singhal et al. 2015), a phenomenon also observed in yeast (Lam and Keeney 2015). This apparent conservation in the placement of recombination sites genome-wide contrasts with widespread variation in the frequency of recombination events across different species of birds. Furthermore, while linkage maps across the genome revealed that recombination rates vary substantially at the fine scale, both LD-based maps and linkage maps showed that recombination events localize to specific genomic features of default open chromatin, such as transcription start sites (TSS), transcription stop sites (TES), and GC-rich sequences including CpG islands (CpGi) (Backström et al. 2010; Kawakami et al. 2014, 2017; Singhal et al. 2015; Weng et al. 2019). In addition, recombination rates were reported in positive association with specific genomic features, including transposable elements (TEs) in flycatchers (Kawakami et al. 2017), which contrasts with observations in plants, where a negative association was reported in Arabidopsis and rice (Rizzon et al. 2002; Shen et al. 2017).

Here, we characterize the fine-scale historical recombination map of an iconic avian system, the Eurasian blackcap (*Sylvia atricapilla*). This migratory songbird species is a powerful model to investigate the migratory behavior of natural populations in an evolutionary framework. A strong heritable component has been demonstrated for migratory traits (e.g. Helbig 1991; Berthold et al. 1992), which makes this species a perfect study system to characterize the underlying molecular machinery modulating behavioral variability (Helbig 1991; Berthold et al. 1992; Liedvogel et al. 2011; Merlin and Liedvogel 2019). The genes and regulatory pathways that shape this iconic seasonal behavior remain enigmatic. Determining the genetic basis of a complex behavioral trait requires fine-scale mapping studies for which a high-resolution recombination map is essential.

Our study aims to (i) characterize the population-scaled historical recombination map for wild-caught blackcap individuals, (ii) assess variability of recombination rates across the genome, and (iii) analyze variation in recombination rates across the genomes concerning specific genomic features such as chromosome size, GC content, density of genes and CpGi, functional elements, and different families of retrotransposons (RTs). Finally, we (iv) evaluate the conservation of this recombination map in a comparative framework, including data from the blackcap's closest sister species, the garden warbler, *Sylvia borin*. We focus on Eurasian blackcaps to assess fine-scale recombination rates and their association with genomic features to generate a deeper understanding of this wild songbird species. This also further elucidates recombination rate patterns in an organism lacking a PRDM9 ortholog.

Our study overcomes many of the limitations of previous studies. Specifically, we utilize high mapping efficiencies leveraging a high-quality chromosome-level genome from wild-caught individuals’ WGS data (for details on the reference genome, see Ishigohoka et al. (2023)). Furthermore, we apply an LD-based approach that considers past demographic fluctuations, taking population sizes at different breakpoints of the focal population into account to alleviate potential bias in inferring population-scaled recombination rates.

## Results

### Inter- and Intrachromosomal Recombination Rate Variation

**Fig. 1.**
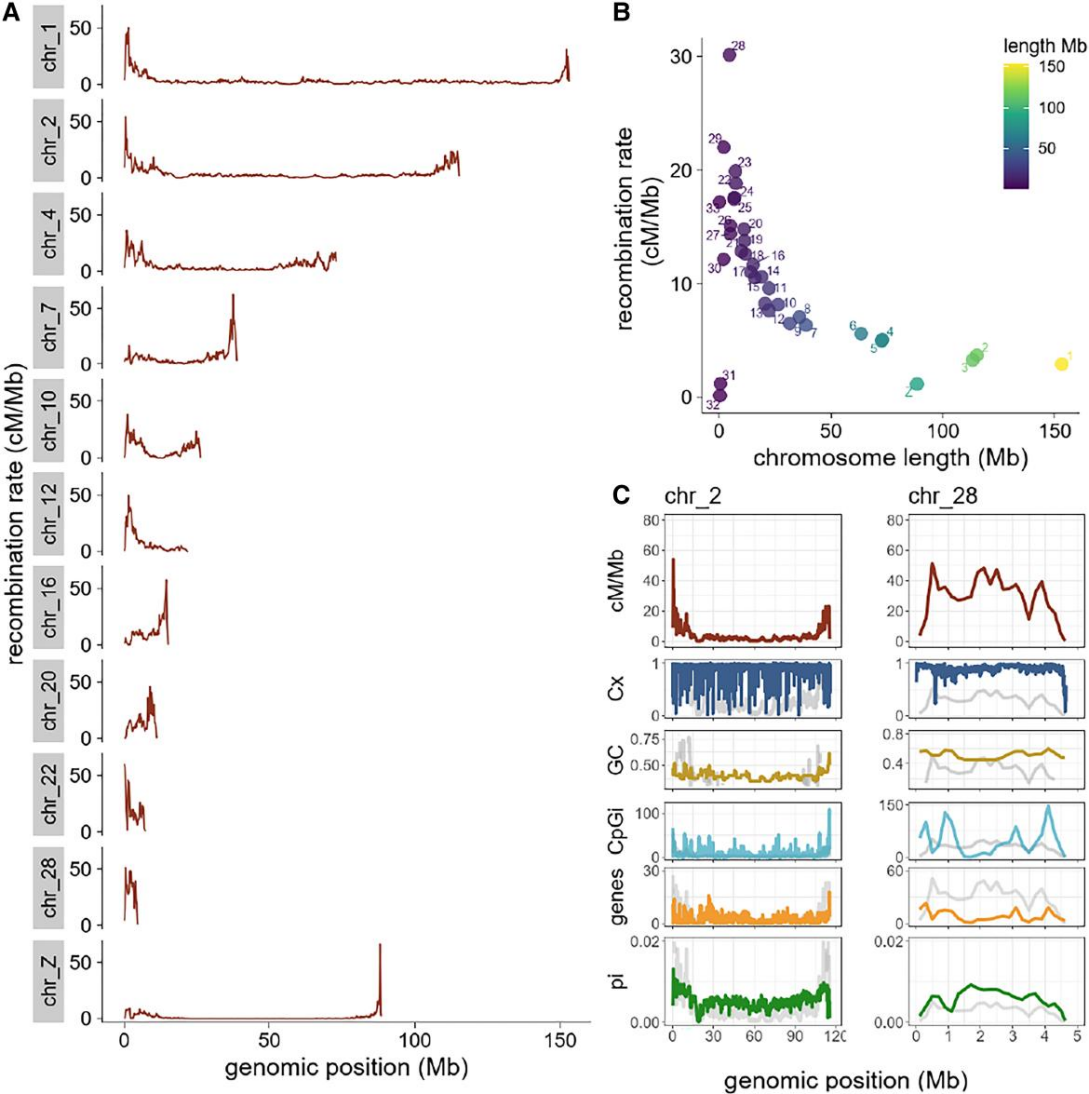
Historical recombination map across the genome and within chromosomes in the Eurasian blackcap. A) Recombination rates calculated in 200 kb windows across genomic positions in a subset of chromosomes representative of different sizes. B) The relationship between chromosome size and recombination rate was measured by Kendall's rank correlation coefficient (τ = −0.6, *P* = 4.7e^−08^). C) Distribution of recombination rates and genomic features (from top to bottom: complexity Cx, GC content, CpGi density, gene density, and nucleotide diversity as pi) calculated in 200 kb windows across exemplified chromosomes 2 and 28. Recombination patterns are shown for each chromosome (in light gray for reference).

Population-scaled recombination rate per-site per-generation average was 5.8 cM/Mb across the Eurasian blackcap genome (as shown in supplementary table S2, Supplementary Material online), while the average rho (ρ) across all chromosomes was 0.23 (supplementary table S3, Supplementary Material online). Our characterization of population-scaled recombination landscape in blackcaps revealed a heterogeneous pattern with variation in recombination rates among and within chromosomes (Fig. 1A, supplementary fig. S1, Supplementary Material online), where rates varied substantially between autosomes, with extremes as low as 0.1 and as high as 30 cM/Mb (supplementary table S2, Supplementary Material online). Shorter chromosomes show elevated recombination rates, as the recombination rate significantly increased with decreasing chromosome size (Fig. 1B, Kendall's tau [*r*_τ_] = −0.61, *P* = 4.7e^−8^). Avian genomes contain macro- and microchromosomes and particularly microchromosomes (<20 Mb) revealed almost three-times higher average recombination rates of 13.9 cM/Mb (supplementary table S2, Supplementary Material online) compared with macrochromosomes with an average rate of 4.8 cM/Mb (Welch's 2-sample *t*-tests, *P* = 2.8e^−5^). To aid comparison, recombination rates are intentionally plotted on the same scale in Fig. 1A, but refer to supplementary fig. S1, Supplementary Material online to appreciate the extent of recombination rate heterogeneity on these chromosomes at a more appropriate scale for smaller chromosomes. In most microchromosomes, recombination was elevated along the entire chromosome (Fig. 1A and C, supplementary table S2, Supplementary Material online, SD ± 11.06), yet 2 of the shortest chromosomes, chromosomes 31 and 32, had particularly low recombination rates (Fig. 1B, supplementary table S2, Supplementary Material online). These chromosomes also have lower SNP densities than all other autosomes (8.3 and 3.1 variants/kb on average for chromosomes 31 and 32, respectively, while the minimum average for all other autosomes was 14.3 variants/kb). Macrochromosomes displayed central recombination deserts and elevated recombination rates toward distal chromosome regions, followed by a sharp drop in recombination rate at the very ends (Fig. 1A).

Sex chromosome Z had the lowest average recombination rate (1.15 cM/Mb; Fig. 1A, supplementary table S2, Supplementary Material online). This chromosome further comprises a 65 kb interval with the overall highest recombination rate (163.17 cM/Mb) at 1 chromosome end, located within an 800 kb block of elevated average recombination rate (89.5 cM/Mb) (Fig. 1A). This region may represent the pseudoautosomal region (PAR).

### Recombination Rates Are Strongly Associated with Nucleotide Diversity, GC Content, and CpG Islands

**Fig. 2.**
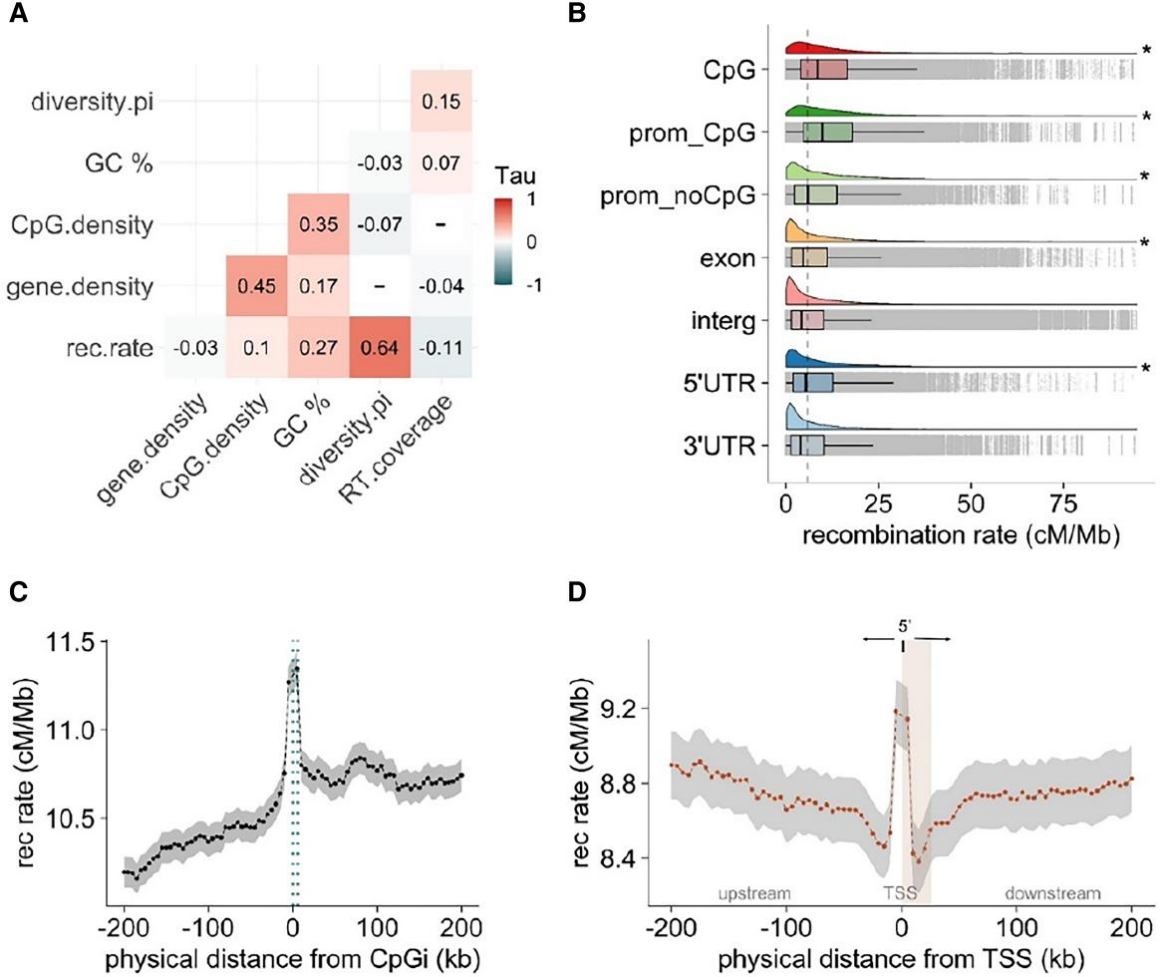
Recombination variation is associated with genomic features and functional elements. A) Genome-wide partial Kendall's rank correlations between recombination rates and genomic features calculated in 200 kb windows. All correlations are significant (*P* < 0.001) except for those marked with (−). B) Cloud plots and box plots show median and average recombination rates in different annotation categories. The color-coded ridgelines (distribution curves) above each box plot show the data distribution for different annotation categories. The dashed line at 5.8 cM/Mb denotes the average genome-wide recombination rate for reference to feature-specific estimates. Features which are annotated with an asterisk (*) indicate a significant deviation of the median recombination, when compared with the genome-wide average recombination rate, assessed using Wilcoxon rank-sum test (with *P* < 0.001). C) Average recombination rate as a function of distance to the nearest CpG island. Dotted lines indicate the start and end of CpG islands. D) Average recombination rates as a function of distance to the nearest TSS; the vertical shadow (in light orange) denotes mRNA of annotated genes. Dots represent the average recombination rate for each 5 kb window, and the shadow (in gray) denotes 95% confidence intervals.

We found a genome-wide strong association between recombination rates and nucleotide diversity (*r*_τ_ = 0.64, *P* < 0.0001) and moderate association with GC content (*r*_τ_ = 0.4, *P* < 0.0001) and CpGi density (*r*_τ_ = 0.3, *P* < 0.0001). These relationships remained significant after performing partial correlations, where all variables were conditioned, as well as when using different window sizes (specifically 50 kb, 100 kb, 200 kb, and 1 Mb) (Fig. 2A, supplementary table S4, Supplementary Material online). Additionally, microchromosomes had significantly higher GC content (*r*_τ_ = −0.84, *P* < 0.001) and density of CpGi (*r*_τ_ = −0.68, *P* < 0.001) (for example, see chromosomes 2 and 28; Fig. 1C, supplementary table S5, Supplementary Material online) compared with macrochromosomes.

Considering that CpGi may play an important role in recombination dynamics and transcription regulation, we further investigated the intrachromosomal distribution of CpGi density and recombination rate. Both showed similar patterns across most chromosomes, and we observed peaks in recombination rates overlapping with CpGi-dense regions (chromosome 2; Fig. 1C, supplementary fig. S3, Supplementary Material online). In some chromosomes, however, we saw contrasting patterns where regions of low recombination were CpGi-rich instead (chromosome 28; Fig. 1C, supplementary fig. S3, Supplementary Material online). In order to decipher whether recombination rates were increased within CpGi or relatively abundant in adjacent regions, we took recombination rates as a function of distance from the nearest CpGi into account. We defined the starting point of each CpGi as zero and calculated recombination rates in 5 kb windows spanning 200 kb regions upstream and downstream of each CpGi. This analysis revealed that recombination increased within the entire CpGi track (upstream: *r*_τ_ = 0.9, *P* < 0.001; downstream: *r*_τ_ = −0.82, *P* < 0.001), flanked by an interval of decreased recombination rates in the respective adjacent up and downstream regions (Fig. 2B).

### High Recombination Rates in Regulatory Regions But Not Necessarily in Gene Bodies

Recombination rates were positively correlated with gene density in our genome-wide pairwise comparison (*r*_τ_ = 0.2, *P* < 0.001; supplementary table S4, Supplementary Material online). However, after controlling for variables such as GC content and CpG density with partial correlation, this association changed to slightly negative (*r*_τ_ = −0.03, *P* < 0.05; Fig. 2A). Shorter chromosomes had significantly higher gene density (*r*_τ_ = −0.96, *P* < 0.001), and intrachromosomal distribution of gene density and recombination rates revealed variable patterns with regions of high recombination in areas of low gene density for some chromosomes (e.g. chromosome 28, Fig. 1C, and chromosomes 16 and 18, supplementary fig. S4, Supplementary Material online). The relationship of recombination with genes or functional elements may depend on gene density, location, and specific regulatory motifs surrounding them and ultimately controlling their expression. These included *cis-*regulatory elements such as promoters and 5′ prime regions, significantly enriched in areas with higher recombination rates (Fig. 2C; Wilcoxon rank test corrected *P* < 2e^−16^). In contrast, recombination rates within intergenic and 3′ untranslated region (UTR) regions were significantly lower than the genome-wide recombination average (Fig. 2C; Wilcoxon test *P* < 0.01). Since recombination rates in organisms lacking PRDM9 are usually elevated in TSS, we evaluate if this was also the case for blackcaps. Hence, we took recombination rates as a function of distance from TSS and confirmed the significant increase of recombination rates in TSS compared with the surrounding regions (upstream: *r*_τ_ = −0.46, *P* = 0.07; downstream: *r*_τ_ = 0.5, *P* = 0.03). In fully annotated genes, a peak of recombination in TSS was followed by a sharp drop in recombination rate in the adjacent downstream region, including gene bodies and 3-UTR regions (Fig. 2D). Similarly, recombination rates in promoters with CpGi were significantly higher than those without CpGi or CpGi regions (Fig. 2C, Wilcoxon rank test corrected, *P* < 2e^−16^). In line with these results, CpGi and gene density showed a strong genome-wide association (Fig. 2A).

### High Recombination Patterns Coincide with Unique Sequences across the Genome

We inferred complexity (Cx) as the uniqueness of sequences across the genome (ranging from 1 representing unique sequences to 0 for repeated sequences). Genome-wide complexity was 0.9, and intrachromosomal distribution of Cx along microchromosomes displayed elevated patterns and few dips, whereas macrochromosomes showed a distinctly more heterogeneous complexity pattern (Fig. 1C, supplementary fig. S5, Supplementary Material online). The between-chromosome comparisons reveal that complexity overall is associated positively with chromosome size (*r*_τ_ = 0.6, *P* = 1.1e^−07^) and negatively with recombination rate (*r*_τ_ = −0.31, *P* = 0.01). However, for the intrachromosomal distribution, we found specific regions with elevated recombination rates overlapping with areas of high complexity (or sequence uniqueness) (Fig. 1C, supplementary fig. S5, Supplementary Material online). We observe drops of complexity in the distal regions in most chromosomes, where recombination rates also decline drastically (supplementary fig. S5, Supplementary Material online). Other regions of low complexity coincide with regions containing few CpGi and low gene density (chromosome 28; Fig. 1C).

### Long Terminal Repeats and Non–Long Terminal Repeat TEs Associate Differently with Recombination Rates

**Fig. 3.**
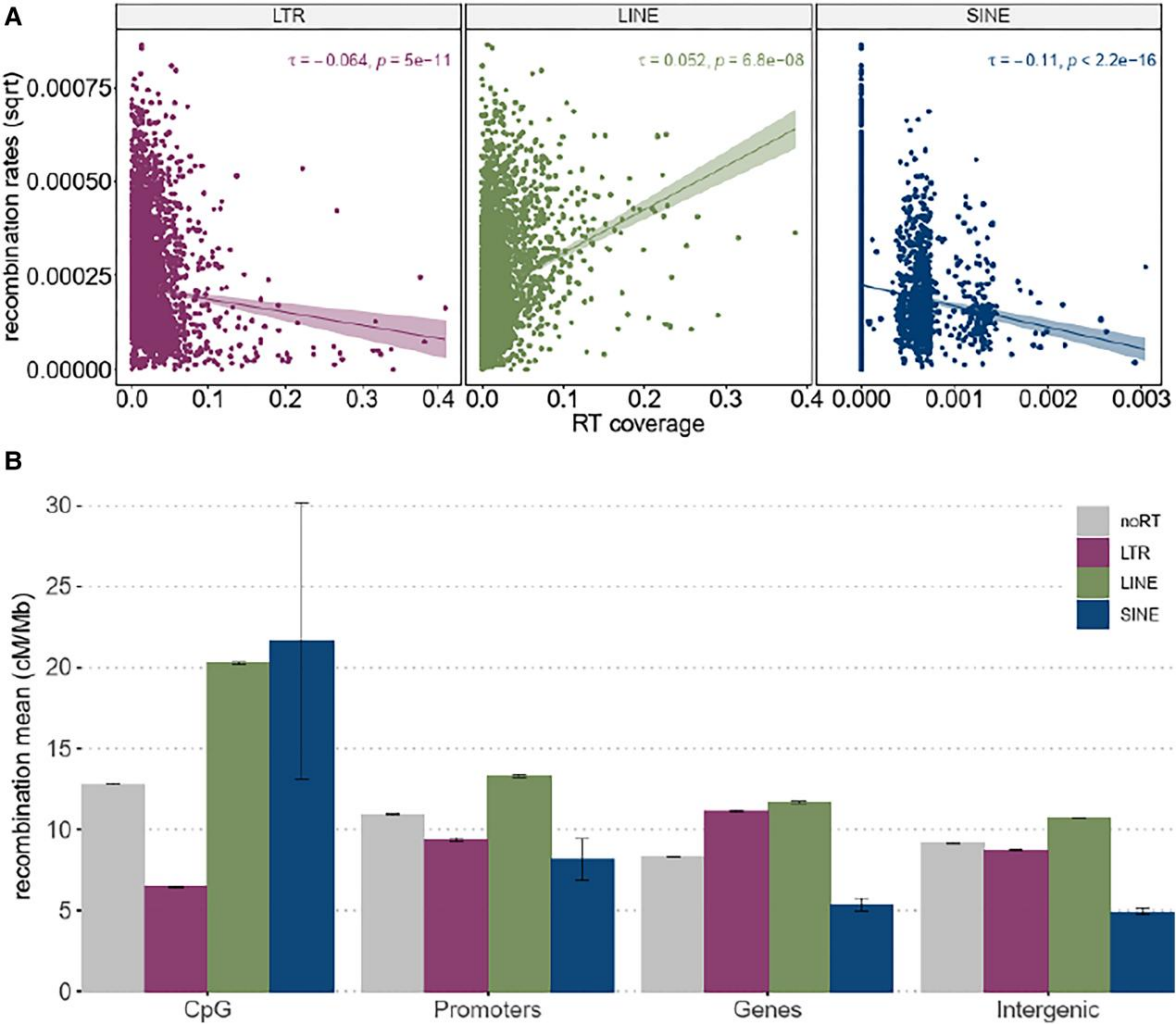
Association between recombination rates and RTs. (A) Correlation between recombination rates (square root transformed) and the coverage of 3 types of RT in 200 kb windows measured with Kendall's tau. B) Bar plots showing the mean recombination rate of different annotation features intersecting with and without (gray) different types of RTs. Errors bars represent 95% confidence intervals. The recombination average in genomic features with RTs significantly differs from genomic features without RTs (Wilcoxon rank-sum test, *P* < 0.05). Average recombination rate of each RT at different genomic features is significantly different (Wilcoxon rank-sum test, *P* < 0.001), except for SINE in genes and intergenic regions.

When we evaluated the association between recombination rates and RT coverage at the genome-wide level in 200 kb windows, recombination rates were not significantly correlated with RT coverage (supplementary table S4, Supplementary Material online). In partial correlations, where GC content, gene density, CpG density, and nucleotide diversity were conditioned, the coefficient turned negative (*r*_τ_ = −0.09, *P* < 0.0001; Fig. 2A). To clarify and further characterize the complex relationship between RTs and recombination, we analyzed different RT families (long terminal repeat [LTR], long-interspersed nuclear element [LINE], and short interspersed transposable element [SINE]) separately, as they have distinct transposition mechanisms and evolutionary histories. Recombination rate and RT coverage were weakly associated and varied between families: LTR and SINES were negatively associated with recombination rate (*r*_τ_ = −0.064, *P* = 5e^–11^ for LTR, *r*_τ_ = −0.11, *P* = 2.2e^−16^ for SINE; Fig. 3A), and LINES showed a weak but significant positive association (*r*_τ_ = 0.052, *P* = 6.8e^–08^; Fig. 3A). Our characterization of RT families across different chromosomes revealed high LTR and LINE (non-LTR) element coverage, particularly at the distal parts of some macrochromosomes where recombination rates are also elevated (supplementary figs. S6 and S7, Supplementary Material online). This pattern was not evident for SINEs (supplementary fig. S8, Supplementary Material online).

To assess whether the presence of RTs contributes to the variation of recombination rates in specific genomic regions, we calculated and compared the median and average recombination rate of CpGi, promoters, genes, and intergenic regions with RTs, including each family separately, and without them. The recombination rate was significantly higher in CpGi overlapping LINEs and SINEs, compared with CpGi alone (Wilcoxon rank-sum test, *P* < 2.2e^−16^ for LINE and *P* < 0.05 for SINE). The recombination rate was significantly elevated in genes containing LTR and LINE elements compared with those without these 2 families of RTs (Wilcoxon rank-sum test, *P* < 2.2e^−16^). Recombination rates within intergenic regions and promoters exclusively associated with LINEs were significantly higher compared with intergenic regions and promoters alone (Wilcoxon rank-sum test, *P* < 2.2e^−16^) (Fig. 3B).

We also investigated each RT family's average recombination rate overlapping with different genomic features. We found that overall, all investigated features associated with LINE elements showed significantly elevated recombination compared with the same features overlapping the other 2 families of RTs (Wilcoxon rank-sum test, *P* < 0.0001; Fig. 3B), except in CpGi, where the average recombination rate of CpGi with SINEs and LINEs was not significantly different (Wilcoxon rank-sum test, *P* = 1; Fig. 3B). LINE and SINE elements located within CpG and promoters had increased recombination rates compared with those located within genes and intergenic regions (Wilcoxon rank-sum test, *P* < 2.2e^−16^; Fig. 3B). In contrast, LTRs in genes showed significantly higher recombination rates compared with LTRs located within intergenic regions, promoters, and CpGi, where recombination rates were generally very low (Wilcoxon rank-sum test, *P* < 2.2e^−16^; Fig. 3B).

### Heterogeneous Conservation of Recombination Rates between Closely Related Sister Species

To assess the similarity of recombination maps between the blackcap and its closest sister species, the garden warbler, recombination rates were estimated for the garden warbler using the same pipeline as was used for the blackcap. The genome-wide average recombination rate in the garden warbler was 2.07 cM/Mb. Similar to the blackcap recombination map, microchromosomes had much higher recombination rates (5.13 cM/Mb on average) compared with macrochromosomes with an average of 1.67 cM/Mb, which also showed deserts in the middle and peaks of recombination at chromosome ends.

**Fig. 4.**
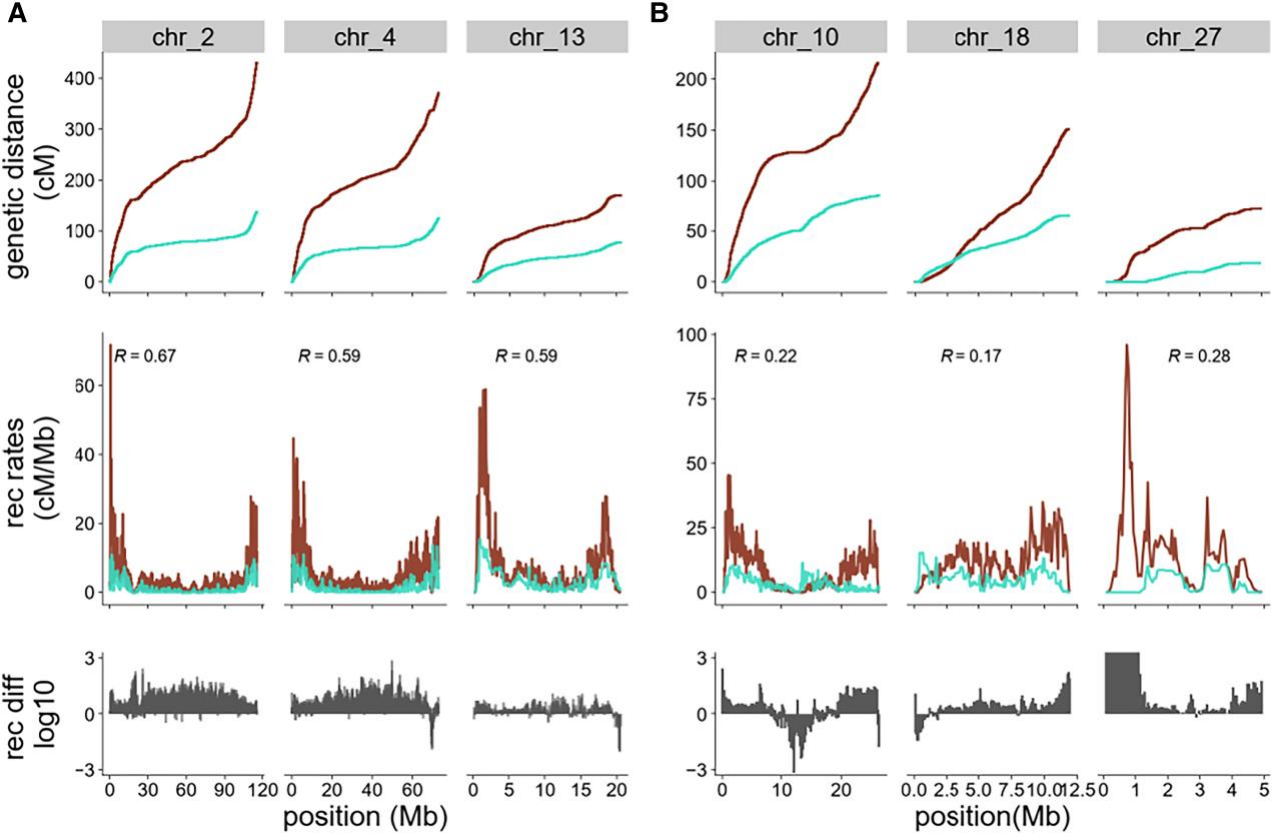
Historical recombination map comparison between closely related sister species. Recombination rates and genetic map for blackcap (dark red) and garden warbler (cyan). Different chromosomes show (A) higher similarity and (B) lower similarity of recombination rates between both species. The first row shows genetic distance in cM, and the middle row shows the distribution of recombination rates, including correlation coefficients measured by Kendall's rank correlation. All comparisons are significant (*P* < 0.001). The third row shows normalized recombination rate difference calculated between both species across different chromosomes.

We compared recombination rates between both species in 50 kb windows to analyze the conservation of these apparent similarities. We found a significant correlation of genome-wide recombination rate (*r*_τ_ = 0.60, *P* = 2.2e^−16^) that increased with wider window sizes (supplementary table S6, Supplementary Material online). We also analyzed the similarity at the level of individual chromosomes, except for some microchromosomes (specifically chr 28, 29, 31, and 32) where SNP density from at least 1 species was insufficient to perform a statistical comparison (supplementary fig. S7, Supplementary Material online). We observed considerable variation in the degree of interspecies correlation between chromosomes. Recombination maps of autosomal chromosomes 2, 4, 6, 9, 11, 13, 19, 20, 21, 23, and 25 showed high similarity between both sister species (Fig. 4A, supplementary figs. S1 and S2, Supplementary Material online). Additionally, recombination rates along chromosome Z are highly similar between species (*r*_τ_ = 0.47, *P* < 2.2e^−16^). Lower correlation coefficients and thus less conservation between species were observed at chromosomes 8, 10, 15, 18, 22, and 27 (Fig. 4B, supplementary figs. S1 and S2, Supplementary Material online). In a subsetted data set, using the same number (*n* = 5) of samples for both species, correlation coefficients were also consistent in both genome-wide (*r*_τ_ = 0.56, *P* = 2.2e^−16^) and intrachromosomal (supplementary fig. S9, Supplementary Material online) recombination map comparisons.

## Discussion

### Genomic and Intrachromosomal Recombination Rate Variation

We show that the historical recombination rates along the blackcap genome are highly variable between and within chromosomes. Our fine-scale recombination rates were inferred from a natural population of blackcaps explicitly accounting for its demographic history and revealed a high average historical population-scaled recombination rate per site per generation of 5.8 cM/Mb in this species. Using the same approach with identical parameters, we estimated the recombination rate of a closely related sister species, the garden warbler, where we found an average population-scaled recombination rate per site per generation of 2.07 cM/Mb. The order of magnitude of the estimated rate for garden warblers matches those reported for other avian species (Singhal et al. 2015; Kawakami et al. 2017).

We observed lower recombination rates across most of the garden warbler genome's chromosomes compared with the blackcap. However, the scaling may not reflect absolute values (Spence and Song 2019), mainly because the mutation rate to recombination rate ratio has not been widely assessed in wild birds. However, relative differences in recombination rate estimates will be robust (Kamm et al. 2016). The difference in average recombination rates may represent differences in scaling or different rates of COs in both species, as well as distinct evolutionary histories, though their correlative nature clearly limits the interpretation of our analyses. Indeed, variation in genome-wide recombination rates differs according to the approach and resolution of our analysis. For example, zebra finches are one of the species with the lowest reported recombination rate, specifically 1.9 cM/Mb in cytological studies (Calderón and Pigozzi 2006). However, contemporary recombination maps based on genetic linkage revealed recombination rates of 2.6 cM/Mb (Stapley et al. 2010) and 1.5 cM/Mb (Backström et al. 2010), and population-scaled recombination rate, estimated using LD-based inference without demography information, reported substantially lower recombination rate of only 0.14 cM/Mb in this finch species (Singhal et al. 2015).

Absolute values of recombination rate must be interpreted with caution. Although our simulations showed that different sample sizes and mutation rates might additionally generate considerable variation in absolute values, recombination patterns and statistical correlations between recombination maps among blackcaps and garden warblers remain robust and consistent. Common recombination patterns found in other bird species were also identified in both species focally studied here, such as elevated recombination rates at the ends and deserts of recombination in the middle of macrochromosomes, supporting a general pattern in birds. This pattern, particularly suppression at the chromosomal center, was also characterized in zebra finches (Backström et al. 2010; Singhal et al. 2015) and may reflect the position of the centromeres, though our analyses are purely limited to in silico approaches. However, heterogeneous recombination landscapes, using whole-genome linkage maps, were also observed in additional passerine species (Kawakami et al. 2014; Hagen et al. 2020; Peñalba et al. 2020; Robledo-Ruiz et al. 2022) as well as in flycatchers and finches, for which the only LD-based historical recombination maps are available (Singhal et al. 2015; Kawakami et al. 2017).

Increased recombination rates toward chromosome ends are a general pattern reported for many bird species (Singhal et al. 2015; Pigozzi 2016; Kawakami et al. 2017; del Priore and Pigozzi 2020), as well as other organisms lacking PRDM9 (Higgins et al. 2012; Lam and Keeney 2015; Yelina et al. 2015; Campbell et al. 2016; Haenel et al. 2018), and even observed in organisms with PRDM9, including humans (Campbell et al. 2016). A spatiotemporal theory explaining the increase in chromosome arms was reported by Higgins et al. (2012) and Haenel et al. (2018), suggesting that recombination initiation occurs at the distal part of the chromosomes, where DSB and CO events occur more often in comparison with the chromosome centers where euchromatin is present. Also, Haenel et al. (2018) propose that high CO rates are primarily given in the chromosomes’ peripheral or distal region. It was further suggested that chromosome homology pairing initiated at the telomeres is slower, resulting in prolonged DSB activity on these chromosomes (Thacker et al. 2014). In support of this idea, Subramanian et al. (2019) revealed that 100 kb regions flanking telomeres continue to receive DSBs even after synaptonemal complex (SC) components down-regulate interstitial recombination initiation in yeast. The high marker density of the blackcap reference genome facilitates the detection of increased recombination rates at the chromosomal ends, even though these regions are of low genome complexity. This further allowed us to overcome some of the limitations of previous studies lacking the high-resolution genomic scaffolding provided by whole-chromosome optical maps and thus often had provided an insufficient resolution at chromosome ends and other highly repetitive regions.

The pattern found on the Z chromosome was distinct from autosomes. Historical recombination rates were low along the entire chromosome except for a specific region at the end. This region may represent the PAR, an area of homology between both sex chromosomes, where homologous recombination can occur in the heterogametic sex. As obligate CO molecules between, ZW is required to enable correct segregation of the sex chromosomes at the meiotic spindle (Mohandas et al. 1992). The characteristic PAR has been characterized in other bird species like flycatcher (Smeds et al. 2014; Kawakami et al. 2017).

### Differential Recombination Rate According to Chromosome Size

Genomic architecture and chromosomal organization influence recombination rate variation. Given that segregation requires an obligatory CO for each chromosome pair, a larger number of recombination events are needed in genomes with higher numbers of chromosomes (e.g. birds) (Fledel-Alon et al. 2009).

Though correlative, our results suggest that high recombination rates in birds stem primarily from regions of highly elevated recombination in microchromosomes with a higher density of functional elements. Elevated recombination rates have been reported for birds and genomes of other taxa with shorter chromosomes, such as fungi, plants (e.g. *Arabidopsis thaliana*), insects (e.g. honeybee, bumblebee) (Haenel et al. 2018), and snakes (Schield et al. 2020). Cytological studies illustrated an increased density of CO in shorter chromosomes, which sufficiently explains the pattern of elevated recombination based on the ratio of CO numbers to chromosome length (Malinovskaya et al. 2018). Genome-wide complexity, defined as sequence uniqueness or nonrepetitiveness, was higher in blackcaps (0.9) compared with humans (0.80) and mice (0.78), where high complexity was also found in functional elements and captured transposon insertions and copy number variation (Pirogov et al. 2019). In our data, the intrachromosomal distribution showed generally elevated complexity patterns in microchromosomes compared with macrochromosomes, yet the interchromosomal association between low complexity and high recombination remained positive. This correlation could be influenced by estimating complexity over the entire chromosome, probably not reflecting fine-scale complexity fluctuations, especially within macrochromosomes.

Moreover, features correlating with high recombination, like promoters and regions upstream of genes, are abundant in tandem repeats in avian genomes, probably lowering the complexity value in those regions. Differences between shorter and longer chromosomes are consistent with findings reported for other bird species, with microchromosomes having a higher density of coding regions and fewer repetitive elements than macrochromosomes (International Chicken Genome Sequencing Consortium 2004; Waters et al. 2021). We also found regions within chromosomes showing low complexity, high abundance of LTRs, and low SNP diversity, which could indicate the location of centromeres because, at least in the human genome, centromeres were indeed located in such stretches of very low complexity (Pirogov et al. 2019). However, with a note of caution, we wish to emphasize that our findings are primarily correlative, causality cannot be directly inferred, and interpretations remain speculative.

### Recombination Variation Associated with Genomic and Annotation Features

Variation in recombination rates was significantly associated with specific genomic features. We confirmed a positive correlation between recombination and GC content, similar to other studies in birds (Singhal et al. 2015; Peñalba et al. 2020) and mammals (Galtier et al. 2001; Smukowski and Noor 2011). It is not yet understood whether high GC content is a cause or a consequence of elevated levels of recombination, as most evidence of GC bias is indirect and comes from observation of GC enrichment at sites with elevated recombination rates. Therefore, high GC content could promote recombination initiation, and recombination promotes GC enrichment via GC-biased gene conversion (gBGC), resulting from DSB repair favoring G:C over A:T nucleotides in high recombining regions. Direct evidence for a gBGC mechanism has thus far only been demonstrated in humans, where it is PRDM9-dependent but can be generated independently of CO (Odenthal-Hesse et al. 2014).

Gene density was positively correlated with recombination rates and slightly negatively correlated in partial correlations. This association might be influenced by the density or proportion of genes and their location in *cis*- or *trans*-regulator elements, affecting their mode of expression, as suggested previously (Peñalba et al. 2020). Indeed, most recombination events did not occur within genes but in regulatory regions such as TSS, promoters, and 5′ UTR regions instead. Earlier studies have also reported an increase in DSBs and COs in distal chromosome regions, potentially due to a prolonged process of chromosome pairing, synapsis, and DSB induction at telomeres (Auton et al. 2013; Choi et al. 2013; Lam and Keeney 2015; Singhal et al. 2015; Kawakami et al. 2017). Our data support the idea that—in the absence of PRDM9—recombination events predominantly occur in functional or regulatory regions.

From an evolutionary perspective, and if recombination primarily reshuffles functional elements in genic regions in organisms lacking PRDM9, recombination might be pivotal for adapting and maintaining the variability of functional elements in the genome. In support of this hypothesis, recombination rates in blackcaps were significantly associated with nucleotide diversity. Similar correlations have previously been found in yeast (Tsai et al. 2010), 3 breeds of chicken (Mugal et al. 2013), and other passerine species (Kawakami et al. 2017). The positive correlation is consistent with the expectations of genomic polymorphism being shaped by linked selection (Burri et al. 2015), particularly background selection, where low recombination regions lead to low diversity.

Our data confirm that CpGi are critical players in shaping recombination rate variation, with CpGi being strongly associated with recombination rates and gene density. Historical recombination events in blackcaps are located primarily in regulatory features containing CpGi, further supporting CpGi as predictors of high recombination. Since CpGi is usually associated with DNA hypomethylation at gene promoters, gene expression, and TSS (Jung and Pfeifer 2013; Angeloni and Bogdanovic 2021), recombination may be linked to regulatory regions and potentially to chromatin modifiers such as H3K4me3 and low levels of DNA methylation (Choi et al. 2013). The contradictory patterns we observed for some chromosomes within regions of high recombination and low CpGi (or the opposite) could possibly be explained by CpGi located outside of functional elements or inactive promoters, where DNA is generally hypermethylated and recombination may be suppressed. Additionally, RTs may alter methylation patterns of CpGi and adjacent regions. Our results indicate that the recombination landscape is not shaped solely by the location of functional elements but may also be influenced by their methylation status.

### The Differential Association between Recombination Rates and RTs

Our results show a differential association between recombination and different types of RTs. Even though the correlation coefficients were weak, this could reflect different scenarios in the dynamic of recombination and the presence of RTs across the genome. In contrast to our findings for the blackcap, a positive association was reported for flycatcher and zebra finch (Kawakami et al. 2017). However, our results show a negative association between recombination and RTs, similar to findings in insects (e.g. *Drosophila*) (Rizzon et al. 2002) and cotton (Shen et al. 2017). As our results demonstrate, the methylation patterns and the age of RTs (Kent et al. 2017; Sultana et al. 2017; Bourque et al. 2018), as well as the type of RTs, can heavily influence the direction of this association, in addition to the mechanism of replication, insertion site affinity, and selective pressures. We found evidence for a bidirectional correlation between recombination and non-LTR elements, with LINE positively and SINE negatively associated with recombination.

The positive association with LINEs could be explained by the fact that RTs can transcribe themselves by targeting upstream regions of genes transcribed by RNA pol III. This suggests that LINE elements would predominantly be found in open chromatin stretches, including functional genetic elements (e.g. promoters), similar to the recombination machinery. Moreover, RTs seem to have an affinity for AT-rich motifs (Lerat et al. 2002; Abrusán et al. 2008), which were associated with elevated recombination in birds and nucleosome-depleted regions in plants and yeast (Jansen and Verstrepen 2011; Choi et al. 2013). For instance, LINE-like RTs form and maintain telomeres in Drosophila, replacing the role of the telomerase enzyme (Pardue and DeBaryshe 2011). Interestingly, we found exceptionally high numbers of RT in the distal part of some chromosomes of the blackcap genome, a pattern also reported for rice (Tian et al. 2009).

Our results demonstrate that the presence of LINEs is associated with the increased local recombination rate in specific genomic features such as promoters, genes, and CpGi, where the recombination rate is already elevated. However, LINEs were also found in intergenic regions where recombination is generally lower, though the average recombination rate in intergenic regions containing these elements was significantly lower compared with other features. Here, intergenic regions contain introns and other genomic sequences located out of genes, which could, at least to some extent, interact with LINEs. Insertion site bias may also explain the low correlation coefficient, as it is widespread in LINEs, particularly in the CR1 subclass, characteristic of birds. In contrast to L1 elements in mammals, high densities of CR1 were found not only in AT-rich but also GC-rich regions where selection might have more efficiently acted on removing insertions from these sites, which can influence the association with recombination rates and differential distribution of LINE families according to age. At least in chicken, young TEs were more predominant and found in regions with high GC content compared with older families of TEs (Abrusán et al. 2008). Similarly, in flycatchers, young retrotransposon subfamilies were also located in high recombining regions (Kawakami et al. 2017).

Recombination rates increase with decreasing SINE coverage, and SINE elements prefer GC sequences (Smit 1999), apparent in our data as the recombination rate in CpGi with SINE was significantly higher compared with other annotation features or CpGi without RTs. This suggests that SINE elements may partially be associated with increased recombination rates in GC-rich sequences and CpGi. The negative association between SINEs and recombination rates may indicate a regulatory role via chromatin repression. This is further supported by our observation that recombination rates in promoters, genes, and intergenic regions with SINEs were significantly lower than regions without these elements. However, methylation patterns and age might influence this interaction drastically, as younger SINE subfamilies show higher methylation than old ones (Pehrsson et al. 2019). Moreover, young Alu elements (a type of SINEs) in humans are randomly distributed along the genome, while old elements with fixed methylation patterns were predominantly found in GC-rich sequences (Smit 1999; Pehrsson et al. 2019). Hence, the methylation patterns of different subfamilies of SINEs could contribute to recombination variation.

We observed a weak negative association of recombination with LTRs and a high coverage distribution of these elements in regions where recombination is low or even suppressed, potentially indicative of centromeres (supplementary fig. S6, Supplementary Material online). In agreement with that, Rizzon et al. (2002) reported LTRs enriched in low gene density, GC content sequences, and low recombination regions like centromeric and pericentromeric regions. Similarly, a study in chicken and rice revealed a negative correlation between LTR densities, recombination, and gene densities (Tian et al. 2009; Mason et al. 2016). Even though we found higher average recombination rates in genes overlapping LTRs compared with genes without them, recombination in promoters and CpG did not increase with the presence of these elements. This suggests a possible scenario where either recombination is boosting selection against RT insertions and further expansion on the genome (Dolgin and Charlesworth 2008) or RTs potentially playing a role in repetitive element maintenance and contributing to recombination suppression.

The genome-wide distribution of RTs, their specific family, replication mechanisms, and insertion sites have consequences on the variation of recombination rates. It remains unclear whether RTs are removed from regions of high recombination through purifying selection or instead accumulated in nonrecombination regions, where selection is weaker (Dolgin and Charlesworth 2008), potentially contributing to recombination suppression (reviewed in Kent et al. 2017). Specific families of RTs in regions of high recombination may play beneficial roles in regulating gene expression or promoter dispersion (reviewed in Pardue and DeBaryshe 2011; Bourque et al. 2018), and selection might not act against them.

In a broader context, and despite being mostly correlative, our study not only characterizes the fine-scale population historical recombination map accounting for past demographic fluctuations (and thus alleviates potential bias in recombination rates inferences for a wild avian system) but also provides an important toolset to focally study sources of selection and other evolutionary forces shaping variation in behavioral traits and population dynamics. Moreover, identifying conserved and nonconserved regions across the recombination maps in very closely related species highlights the importance and potential to expand our characterization of recombination analyses from this focal resident blackcap population to additional populations across the species distribution range, as well as other species, using variable demographic data, selective pressures, and species-specific mutation rates. With the advances in sequencing technology and the availability of a growing number of high-quality bird genomes, the approach applied here may facilitate the characterization of comparable recombination maps in the future. Moreover, we showed the association between recombination rates and genomic features. However, given the primarily correlative nature of our analyses, most interpretations of causality remain speculative and experimental approaches, such as sperm resequencing, hotspot analyses, or cytological assessment, are needed for more direct insight into the evolution and causality of these interactions.

## Materials and Methods

### Samples, Reference Genome, and Annotation

We used WGS data of 19 resident male blackcaps collected from 2 locations in Spain (Cazalla de la Sierra *n* = 11, Gibraltar *n* = 8) previously reported as part of a population genomics analysis (Delmore et al. 2020). Individual WGS data obtained by Illumina NextSeq 500 were mapped against our chromosomal-level reference genome of a female from a resident population in Tarifa, Spain, with a median coverage of 15.1 ± 11.1X (Ishigohoka et al. 2023 and Rhie et al. 2021 for all information on the blackcap reference genome; genome available under NCBI BioProject PRJNA558064, accession number GCA_009819655.1).

Genome annotation was carried out using MAKER v.3.01.02 (Campbell et al. 2014). We ran RepeatMasker v.4.0.9 (Smit et al. 2015) to soft-mask the genome assembly using a library of TEs from the collared flycatcher (Suh et al. 2018) and blue-capped cordon bleu (Boman et al. 2019) as input. We de novo assembled RNA-seq transcriptome data of 3 different brain regions (2 blackcap individuals for each brain region) using Trinity v.2.9.1 (Grabherr et al. 2011). This data was combined with an Iso-seq transcriptome of the brain and reproductive organs from 1 male and 1 female blackcap to serve as evidence for gene prediction. Additionally, cDNA and protein sequences of 3 avian species (zebra finch, chicken, and flycatchers) from Ensembl and TrEMBL, as well as manually curated avian cDNA and protein sequences from RefSeq and Swiss-Prot, were included as further evidence. We used the ab initio gene predictors Augustus v.3.3 (Keller et al. 2011) (species chicken) and SNAP (Korf 2004) to refine the gene predictions. SNAP was trained iteratively on the output of MAKER to improve its accuracy. Only gene predictions with annotation edit distance (AED) ≤ 0.5 are included in our final annotation. We performed functional annotation on the predicted protein sequences using Blast v.2.10.1+ with the Swiss-Prot database and InterProScan v.5.54-87.0 with the Pfam application.

### Mapping and Variant Calling

We used a modified pipeline based on the GATK best practices for short-read sequencing for mapping and variant calling. First, the quality of adapter bases in the sequenced reads of the 19 samples was set to 2 with Picard MarkIlluminaAdapters (v.2.21.9 (http://broadinstitute.github.io/picard), followed by paired-end mapping to the reference using BWA mem (Li 2013). Then, we marked reads as duplicates based on mapping position and insert size since we wanted to keep unmapped read mates and supplementary reads for completeness. To evaluate how well the samples mapped to the reference, we performed quality control of our generated read alignments (BAM files) using QualiMap v.2.2.1 (Okonechnikov et al. 2016); Picard CollectMultipleMetrics, CollectRawWgsMetrics, and CollectWgsMetrics; and MultiQC v.1.8 (Ewels et al. 2016). We used GATK v.4.1.6.0 HaplotypeCaller to call SNPs per sample and output them as gVCF files. Afterwards, the 19 gVCF files were combined using GATK CombineGVCFs. From the gVCF file with 19 samples, we called variants using GenotypeGVCFs. SNPs were selected using GATK SelectVariants and hard-filtered with the following criteria: QD < 2.5, FS > 45.0, SOR > 3.0, MG < 40, MQRankSum < −12.5, and ReadPosRankSum < −8.0. Finally, we selected 19,917,215 variants for the recombination inference in the blackcap by applying a Genotype Quality filter of 20, a minimum depth of 5, a maximum depth of 60, and a missingness of 0.7 with VCFtools v.4.0 (Danecek et al. 2011). Furthermore, we removed singletons (minimum allele frequency > 0.03) using VCFtools and GATK SelectVariants, as nonreproducible singletons would include most randomly distributed sequencing errors that could bias LD analyses. We extracted scaffolds and chromosomes by taking only biallelic sites using GATK SelectVariants. We only considered reads mapping to chromosomes for the recombination estimation and further analysis. In addition to the 19 individual blackcap WGS data, we also included WGS data for 5 individuals (4 males, 1 female) of the closely related sister species, the garden warbler. For these samples, the VCFs with hard-filtered SNPs specific for the garden warbler were taken from Ishigohoka et al. (2023). We further filtered these SNPs precisely as previously described for the blackcap, yielding 11,386,509 SNPs for further analysis in the garden warbler.

### Inferring Historical Recombination Rates across the Blackcap Genome

We used Pyrho, which can take unphased data, to estimate population-specific recombination rates per generation and characterized their genome distribution (Kamm et al. 2016; Spence and Song 2019). The program relies on a composite-likelihood approach, considering population-specific demography to compute lookup tables and calculate the optimal parameters for the estimation.

Pyrho estimates the recombination rate “rho = 4Ne *r*,” which is then scaled to the per generation per site recombination rate (*r*) using demography (the effective population sizes, Ne) as well as specified mutation rate. We used the mutation rate of the collared flycatcher, another songbird species (4.6 × 10^−09^ site/generation) (Smeds et al. 2016), assuming that closely related bird species would have a similar mutation rate. Initially, Pyrho precomputes likelihood tables under a demographic model, accounting for population size fluctuations across time and the mutation rate. This was achieved by the “make_table” option, which took a series of population sizes and breakpoints of the focal continental resident population inferred by MSMC2 (Schiffels and Wang 2020) as part of a precursor study in blackcaps (see Delmore et al. (2020) for full description) and the respective mutation rate as input. After running “hyper-param” in Pyrho, we found a block penalty of 20 and a window size of 50 kb to give optimal resolution in recombination rate estimates for our data set. We then inputted unphased genotypes for each individual in VCF format and estimated recombination rates with Pyrho “optimize” for each chromosome separately. The estimates of Pyrho (scaled recombination per site per generation rate) were converted to cM/Mb using a custom Unix script (https://github.com/Karenbc/Recombination-rates-and-genomic-features-Blackcap/blob/main/Recombination_rate_estimation/Conversion_OutPyrho_to_cM_Mb_extended.sh), which we also used to output the linkage genetic map (genetic distance in cM).

To compare resolution, we first calculated population-scaled recombination rates in different sizes of nonoverlapping windows (50 kb, 100 kb, 200 kb, and 1 Mb). We used a custom Python script (https://github.com/Karenbc/Recombination-rates-and-genomic-features-Blackcap/blob/main/Recombination_rate_estimation/PyrhOut_wtAvg_rates_windows.sh) to calculate the average rates weighted by the physical distance between each pair of sites where recombination was estimated. We used the “ggplot2” package in R software to plot recombination rates against the physical distance (R Core Team 2021; Wickham 2016).

To determine the expected number of COs for each chromosome, we multiply each chromosome’s average per-site per-generation recombination rate by chromosome lengths. We took the sum of all expected COs as the number of expected COs per generation for the entire genome, analogous to the method used (Spence and Song 2019). Additionally, we calculated rho (p) for each chromosome by multiplying the average recombination rate per site per generation (“*r*”) with 4 Ne. To infer effective population size (Ne), we divided time-averaged theta (*θ*) by mutation rate (*µ*). Each chromosome's time-averaged theta (*θ*) was calculated by average pairwise heterozygosity (*π*), scaled to the number of callable sites. These data result from estimates of nucleotide diversity described in Characterization of Recombination Rate Variation with Respect to Genomic Features. We used a per-generation mutation rate (*µ* = 4.6 × 10^−9^) from the closely related species collared flycatcher (Smeds et al. 2016).

### Testing for Association between Recombination Rates and Genome Complexity

To assess the association of recombination rates with reference genome complexity, a measure of the uniqueness of sequences across the genome, we used “Macle,” a program that estimates match complexity of sequence strings genome-wide (Pirogov et al. 2019). This program outputs values of 1 for all unique sequences and values as low as 0 for sequences repeated multiple times. Complexity was initially calculated for the whole genome and each chromosome separately. We then calculated and compared complexity maps across the genome using different overlapping window sizes (10, 50, and 200 kb). Analogous to Pirogov et al. (2019), the best resolution was achieved for a window size of 10 kb, which was then chosen for final calculations of genome complexity for each chromosome.

### Characterization of Recombination Rate Variation with Respect to Genomic Features

To evaluate the genome-wide association between recombination rates and selected genomic features, we estimated GC content using GC-Profile (Gao and Zhang 2006) and calculated the weighted averaged GC content in 200 kb and 1 Mb nonoverlapping windows considering the length of each sequence where GC was inferred. CpG islands, defined as DNA stretches with high CG content (usually >50%) and frequent absence of DNA methylation (Jung and Pfeifer 2013), were identified using a distance-based algorithm implemented in CpGcluster v1.0 (Hackenberg et al. 2006) setting a minimum length of at least 50 bp and maximal *P*-value of 1E^−5^. Density and coverage of CpGi and genes were calculated by counting the number of elements and calculating their fraction within 200 kb and 1 Mb nonoverlapping windows using bedtools “annotate” (Quinlan 2014). We calculated nucleotide diversity (*π*) using “all sites” VCF and varying tools from the “genomics general” toolkit (Martin et al. 2020). We used parseVCFs.py (https://github.com/simonhmartin/genomics_general/tree/master/VCF_processing release 0.4) to filter the “all sites” VCF similarly to the variant VCF, with min-depth >5 and max <60. We then ran popgenWindows.py (https://github.com/simonhmartin/genomics_general release 0.4) with a window size of 200 kb to ensure a minimum of 20 kb called in each window. As our missingness equivalent, we set the proportion of individuals with at least 20 kb per window to be 0.7 (-minData). Considering recombination rates are usually exponentially distributed, Kendal's rank correlation test with a significance threshold of 5% was used for all the pairwise and partial correlations in 200 kb and 1 Mb windows performed in R with the “ggcorrplot” (https://github.com/kassambara/ggcorrplot) and “ppcor” package (Kim 2015), respectively. We controlled for nonpredictor variables in partial correlation analyses by including GC content, CpGi, gene density, nucleotide diversity, and TEs as potential confounding variables.

For the interchromosomal comparisons, we first estimated the GC content of each chromosome separately using “Geecee” in EMBOSS (Rice et al. 2000) and calculated gene and CpGi density as the number of elements divided by the length of each chromosome. Then, these variables were correlated with weighted average recombination rates per chromosome in cM/Mb, equivalent to genetic map distance divided by chromosome length in Mb. In addition, the high quality of our reference genome with chromosome resolution facilitated measuring the association between chromosome length (measured in Mb and log-transformed) with recombination rates and genomic features separately using Pearson's correlation coefficient.

To investigate variation of recombination rates concerning specific genomic functional elements in the blackcap genome, we assigned different annotation categories: exons, intergenic regions, untranslated upstream and downstream regions surrounding the mRNA (5′ UTR, 3′ UTR), TSS (first position of annotated mRNA), and promoters (defined as 2 kb upstream of TSS) with and without CpGi. Then, recombination rates (in cM/Mb) were intersected with all genomic features using bedtools “intersect,” and the average recombination rate was calculated for each annotation category. We performed the Wilcoxon rank-sum test with multiple test groups and visualized it with the “ggplot2” package in R. Additionally, we calculated recombination rates as a function of distance from TSS and CpGi. We used bedtools and assigned 0 as the first position to the closest gene and CpGi, respectively. Next, we calculated the average recombination rate in 5 kb nonoverlapping windows spanning 200 kb upstream and downstream, considering the direction of genes in case of TSS. The same was performed for CpGi. We plotted and performed statistics using R and the package “ggplot2.” Additionally, we calculated correlations using Kendall's rank correlation test in R with “cor.test” function between the 50 kb upstream and downstream distance from TSS and CpGi with averaged recombination rates, respectively. Correlation tests for upstream and downstream distances were performed separately.

### Characterizing the Association between Recombination Rates and RTs

To evaluate the association between recombination rates and RTs, we utilized the annotation of RTs described in Bours et al. (2022). In brief, the prediction of transposons was calculated using RepeatModeler v.1.0.11 (Bao et al. 2015); LTRs were predicted using LTRharvest (Ellinghaus et al. 2008) and LTR-related HMM (Hidden Markov Model) (Mistry et al. 2021). Transposons were then annotated with RepeatMasker v.4.1.0 (Smit et al. 2015) using manually curated repeat libraries of 2 bird species.

We classified RTs into 3 families: (i) LTRs and 2 subclasses of non-LTR RTs, (ii) LINEs and (iii) SINEs. We calculated density and coverage for all RTs together and each family separately, using 200 kb windows, and measured the correlation between recombination rates (square root transformed) using Kendall's rank correlation test in R. Coverage was used for the analysis since some RTs in the automated pipeline were fragmented, and using density instead of coverage could skew the association of these RTs.

To assess whether the presence of RTs is associated with recombination rate variation in specific annotation features, we calculated and compared median and average recombination rates of CpGi, genes, promoters, and intergenic regions with (overlapping) and without (nonoverlapping) any of the 3 RTs families. We additionally compared average recombination rate of each RT family overlapping with different genomic features to characterize the association between TEs and recombination rates along the genome. We conducted unpaired 2-sample Wilcoxon rank-sum tests (Mann–Whitney test) for each genomic feature and each RT family separately.

### Evaluating the Conservation of Recombination Maps between the Blackcap and Garden Warbler

To evaluate the conservation of historical recombination rates, we compared recombination maps of the blackcap with its closest sister species, the garden warbler. To estimate recombination rates in the garden warbler, we used Pyrho following the same pipeline as for the blackcap (Materials and methods) on an unphased VCF file with SNPs of 5 individuals. We used garden warbler demography estimated with MSMC2 from Ishigohoka et al. (2023) and compared recombination rates between both species in 50 kb, 100 kb, 200 kb, and 1 Mb nonoverlapping windows. We performed Kendall's rank correlation test genome-wide and within chromosomes using cor.test function in R and ggplot2 for visualization. Finally, we calculated recombination rate ratio between both species by subtracting the log-transformed recombination rate calculated in 50 kb windows.

Given the difference in sample size between blackcap (*n* = 19) and garden warbler (*n* = 5), we wanted to assess the correlation of recombination rate between these species using identical sample sizes. For that, we subsetted 5 blackcap samples and estimated recombination rates with the same pipeline used previously with a block penalty of 20 and a window size of 50. We calculated recombination rates in 50 kb windows and estimate correlations genome-wide and within chromosomes, as described previously, with Kendall's rank correlation test.

## Supplementary Material

Supplementary material is available at *Genome Biology and Evolution* online.

## Supplementary Material

evad233_Supplementary_Data

## Data Availability

The primary and alternate haplotype assemblies for the Eurasian blackcap can be found under NCBI BioProject PRJNA558064, accession numbers GCA_009819655.1 and GCA_009819715.1 (Ishigohoka et al. 2023). All scripts used for our analyses were uploaded to GitHub (https://github.com/Karenbc/Recombination-rates-and-genomic-features-Blackcap). The recombination data set and genome annotation are deposited at Zenodo (https://zenodo.org/records/10234492).
