# Supplementary material for: Fine-Scale Map Reveals Highly Variable Recombination Rates Associated with Genomic Features in the Eurasian Blackcap
Source: Genome Biol Evol. 2024 Jan 10;16(1):evad233. doi: 10.1093/gbe/evad233 (PMC10781513; doi:10.1093/gbe/evad233)
Supplement: evad233_Supplementary_Data [file evad233_supplementary_data.docx]

**Supplementary Material**

**Table S1.** Contemporary and historical recombination rate studies in bird species. For cytological estimates the average number of MLH1 foci in oocytes and spermatocytes and the corresponding scaled cM/Mb is reported. Linkage based studies are reported with the estimated recombination rate in cM/Mb, the map length in cM, genome size in Mb, and the density of markers. For historical recombination studies the genome-wide average recombination rates in cM/Mb, information about demography usage for the estimation, the genome size in Mb, and the density of markers.

* based on the assumption that great tit and blue tit have a comparable genome size

** values determined based on dividing the map length in cM by genome size in Mb

**٭**recombination rates per-site per-generation in the Eurasian blackcap

**TableS2.** Chromosomes lengths, weighted average, and SD recombination rate per chromosome for the Eurasian blackcap and the closely related garden warbler. Recombination rates were inferred using Pyrho with a block penalty of 20 and window size 50 kb.

|  |  | **Eurasian Blackcap** | | | | | **Garden warbler** | | | | |
| --- | --- | --- | --- | --- | --- | --- | --- | --- | --- | --- | --- |
| Chromosome | Length (Mb) | Genetic Map  (cM) | Rec mean  (cM/Mb) | | | Rec SD  (cM/Mb) | | | Genetic Map  (cM) | Rec mean  (cM/Mb) | Rec SD  (cM/Mb) |
| 1 | 153,2 | 446,3 | | 2,9 | 6,2 | | | 144,5 | | 0,94 | 1,8 |
| 2 | 115,3 | 428,9 | | 3,7 | 7,3 | | | 137,8E | | 1,19 | 2,3 |
| 3 | 113,4 | 372,6 | | 3,3 | 6,5 | | | 108,5 | | 0,96 | 2,2 |
| 4 | 73 | 370,5 | | 5,1 | 7,8 | | | 124,6 | | 1,71 | 2,8 |
| 5 | 72,6 | 363,1 | | 5,0 | 8,6 | | | 117,2 | | 1,61E | 2,8 |
| 6 | 63,4 | 355,5 | | 5,6 | 8,0 | | | 107,2 | | 1,69E | 2,4 |
| 7 | 38,9 | 246,7 | | 6,4 | 8,7 | | | 79,5 | | 2,05E | 2,6 |
| 8 | 36 | 254,5 | | 7,1 | 7,9 | | | 78,7 | | 2,19 | 2,3 |
| 9 | 31,7 | 206,9 | | 6,5 | 10,8 | | | 90,9 | | 2,87 | 3,0 |
| 10 | 26,4 | 215,8 | | 8,2 | 9,1 | | | 85,6 | | 3,24 | 2,8 |
| 11 | 22,3 | 214,3 | | 9,6 | 9,1 | | | 87,3 | | 3,91 | 2,7 |
| 12 | 22,2 | 170,6 | | 7,7 | 11,6 | | | 81,6 | | 3,67 | 3,1 |
| 13 | 20,6 | 170 | | 8,3 | 11,1 | | | 76,9 | | 3,74 | 3,1 |
| 14 | 19,1 | 202,4 | | 10,6 | 9,7 | | | 73,8 | | 3,87 | 3,0 |
| 15 | 16,1 | 168,7 | | 10,5 | 11,4 | | | 74,0 | | 4,62 | 1,6 |
| 16 | 15,2 | 177,7 | | 11,7 | 12,3 | | | 71,4 | | 4,69 | 2,4 |
| 17 | 14,2 | 156,3 | | 11,0 | 10,2 | | | 66,8 | | 4,71 | 3,0 |
| 18 | 12 | 151 | | 12,6 | 8,7 | | | 65,5 | | 5,46 | 3,3 |
| 19 | 11,4 | 156,9 | | 13,8 | 10,8 | | | 68,7 | | 6,05 | 3,0 |
| 20 | 11,2 | 166,5 | | 14,8 | 11,2 | | | 59,1 | | 5,26 | 2,7 |
| 21 | 10,1 | 129,3 | | 12,9 | 9,5 | | | 60,1 | | 5,99 | 2,7 |
| 22 | 7,6 | 142,7 | | 18,8 | 14,5 | | | 35,5 | | 4,71 | 2,9 |
| 23 | 7,5 | 148,8 | | 19,9 | 13,5 | | | 53,3 | | 7,14 | 3,1 |
| 24 | 6,9 | 120,7 | | 17,6 | 8,8 | | | 52,3 | | 7,64 | 3,3 |
| 25 | 6,8 | 118,3 | | 17,5 | 13,6 | | | 48,1 | | 7,15 | 3,7 |
| 26 | 5,1 | 76,2 | | 15,1 | 11,1 | | | 25,3 | | 5,01 | 4,1 |
| 27 | 5 | 72 | | 14,4 | 15,5 | | | 18,4 | | 3,70 | 3,9 |
| 28 | 4,7 | 140,2 | | 30,2 | 18,7 | | | 18,7 | | 4,03 | 5,3 |
| 29 | 2,2 | 48,4 | | 22,0 | 17,6 | | | 10,3 | | 4,71 | 4,0 |
| 30 | 2 | 24,5 | | 12,2 | 11,5 | | | 7,6 | | 3,83 | 2,1 |
| 31 | 0,6 | 0,7 | | 1,2 | 1,8 | | | 0,04 | | 3,8 | 0,4 |
| 32 | 0,4 | 0,1 | | 0,1 | 0,4 | | | 0,03 | | 0,06 | 0 |
| 33 | 0,3 | 5,1 | | 17,2 | 10,5 | | | 0,59 | | 2 | 0 |
| Z | 88,6 | 102 | | 1,2 | 7 | | | 11,6 | | 0,13 | 0,4 |

**Tale S3.** The potential number of crossovers per chromosome occurring at each generation and the average nucleotide diversity (pi). Recombination rate in Pyrho is estimated as 4Ne*r and then re-scaled to the per-generation per site recombination rate (r) using the specified mutation rate. Average recombination rate for each chromosome in the blackcap genome (ρ, rho). is also shown. The shortest micro-chromosome 31, 32, 33 were excluded due to the lower SNP density.

| **Chromosome** | **pi** | **crossover/**  **generation** | **ρ = 4Ner** |
| --- | --- | --- | --- |
| 1 | 0,00656 | 4,4 | 0,042 |
| 2 | 0,00707 | 4,3 | 0,058 |
| 3 | 0,00651 | 3,7 | 0,047 |
| 4 | 0,00813 | 3,7 | 0,092 |
| 5 | 0,00760 | 3,6 | 0,085 |
| 6 | 0,00748 | 3,5 | 0,093 |
| 7 | 0,00726 | 2,5 | 0,104 |
| 8 | 0,00753 | 2,5 | 0,120 |
| 9 | 0,00718 | 2,1 | 0,104 |
| 10 | 0,00733 | 2,2 | 0,132 |
| 11 | 0,00825 | 2,2 | 0,182 |
| 12 | 0,00746 | 1,7 | 0,128 |
| 13 | 0,00713 | 1,7 | 0,131 |
| 14 | 0,00826 | 2,0 | 0,196 |
| 15 | 0,00859 | 1,7 | 0,199 |
| 16 | 0,00838 | 1,8 | 0,217 |
| 17 | 0,00828 | 1,6 | 0,205 |
| 18 | 0,00969 | 1,5 | 0,283 |
| 19 | 0,00866 | 1,6 | 0,274 |
| 20 | 0,00814 | 1,6 | 0,266 |
| 21 | 0,00936 | 1,3 | 0,265 |
| 22 | 0,00911 | 1,4 | 0,388 |
| 23 | 0,00956 | 1,5 | 0,429 |
| 24 | 0,00994 | 1,2 | 0,388 |
| 25 | 0,00989 | 1,2 | 0,405 |
| 26 | 0,00778 | 0,7 | 0,258 |
| 27 | 0,00838 | 0,7 | 0,262 |
| 28 | 0,01150 | 1,4 | 0,796 |
| 29 | 0,00945 | 0,5 | 0,563 |
| 30 | 0,00857 | 0,3 | 0,258 |

**Table S4.** Genome-wide pairwise correlations and partial correlations between recombination rates with genomic features calculated in 200 kb and 1 Mb windows.

| **Kendall's tau correlation coefficients** | | | | |
| --- | --- | --- | --- | --- |
|  | **Pairwise correlation** | | **Partial**  **correlation** | |
|  | **200 kb** | **1 Mb** | **200 kb** | **1 Mb** |
| CpG density | 0,3*** | 0,4*** | 0,1*** | 0.13*** |
| Gene density | 0,2*** | 0,3*** | -0,03* | ns |
| GC content | 0,4*** | 0,4 *** | 0,27*** | 0,27*** |
| RT density | 0,1*** | 0,1*** | ns | 0,12*** |
| RT coverage | ns | ns | -0,09*** | 0,1***' |
| ***p<0.001, **p<0,01, *p<0,05, ns= non-significant p>0.05 | | | | |

**Table S5.** Intra-chromosomal comparison. Pearson correlations between chromosome length and the average recombination rates per chromosome with CpGi density, gene density, and GC content calculated for each chromosome. Kendall’s tau correlation between chromosome length and the average recombination rates per chromosome with complexity (Cx).

| Inter-chromosomal comparisons: Pearson’s correlation coefficients | | | |  |
| --- | --- | --- | --- | --- |
|  | **CpG density** | **Gene density**  **(logT)** | **GC content** | **Complexity**  Kendall tau |
| **Chr_Length (logT)** | -0,68 *** | -0,96 *** | -0,84*** | 0,6*** |
| **Rec cM/Mb** | 0,68 *** | 0,53 ** | 0,66*** | -0,31* |
| ***p<0.001,**p<0.01 |  |  |  |  |

**Table S6.** Recombination rates comparison between the blackcap and garden warbler genome estimated for different window size.

| **Window size** | **Kendall’s tau coefficient** |
| --- | --- |
| 50 kb | 0,6083 *** |
| 100 kb | 0,6274 *** |
| 200 kb | 0,6469 *** |
| 1 Mb | 0,7115 *** |
| ***p < 0.001 |  |

**Figure S1** Distribution of recombination rates calculated in 50 kb non-overlapping windows in the blackcap (dark red) and garden warbler (cyan) across all chromosomes. Kendall non-parametric correlation coefficients reported for each chromosome.


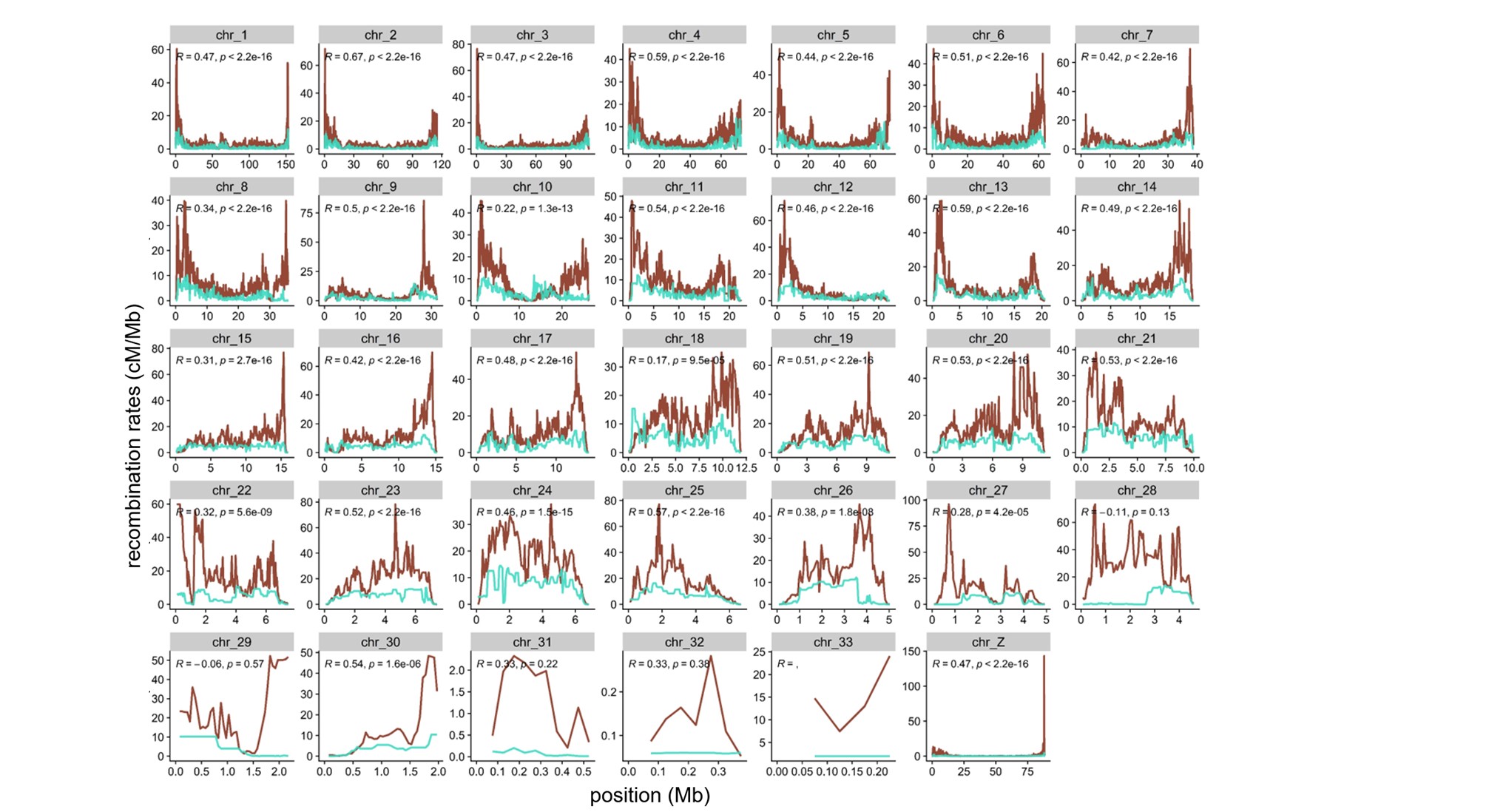


**Figure S2** Genetic map of the blackcap (SylAtr, dark red) and garden warbler (SylBor, cyan) across all chromosomes.


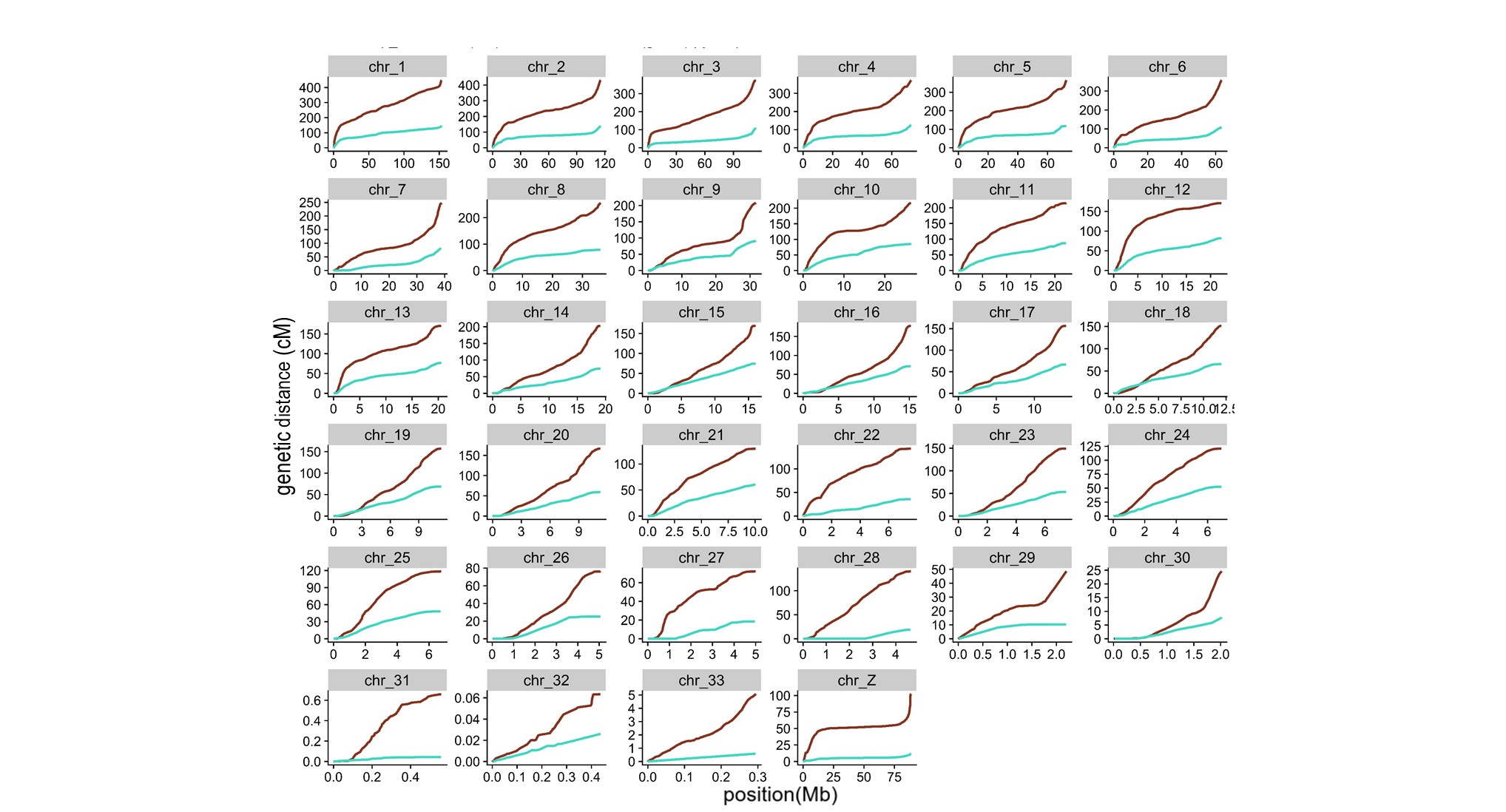


**Figure S3**. Distribution of CpGi density (right axis, turquois) and recombination rates in 200 kb windows (left axis; black) across all chromosomes.


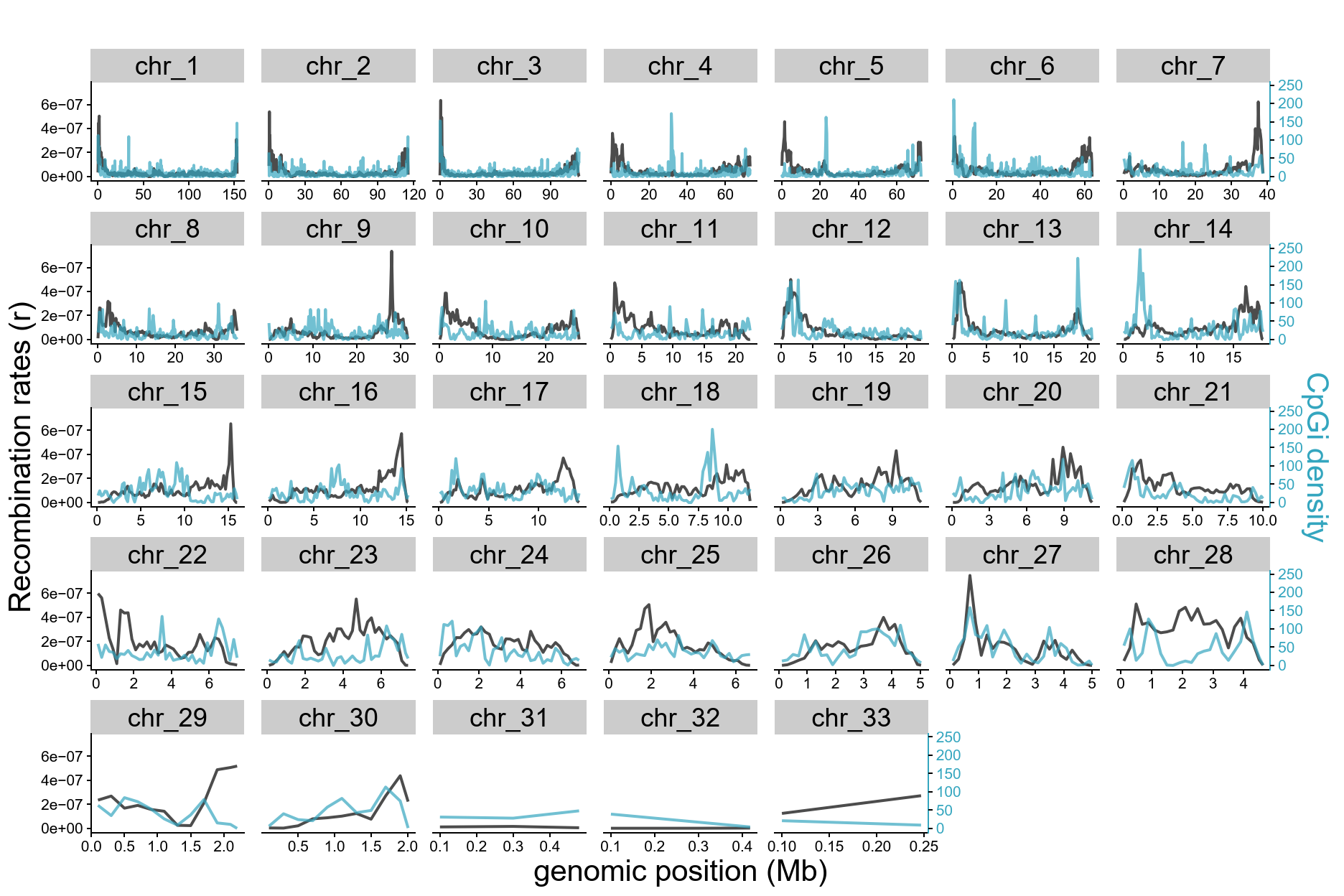


**Figure S4** Distribution of gene density (right axis; orange) and recombination rates in 200 kb windows (left axis; black) across all chromosomes.


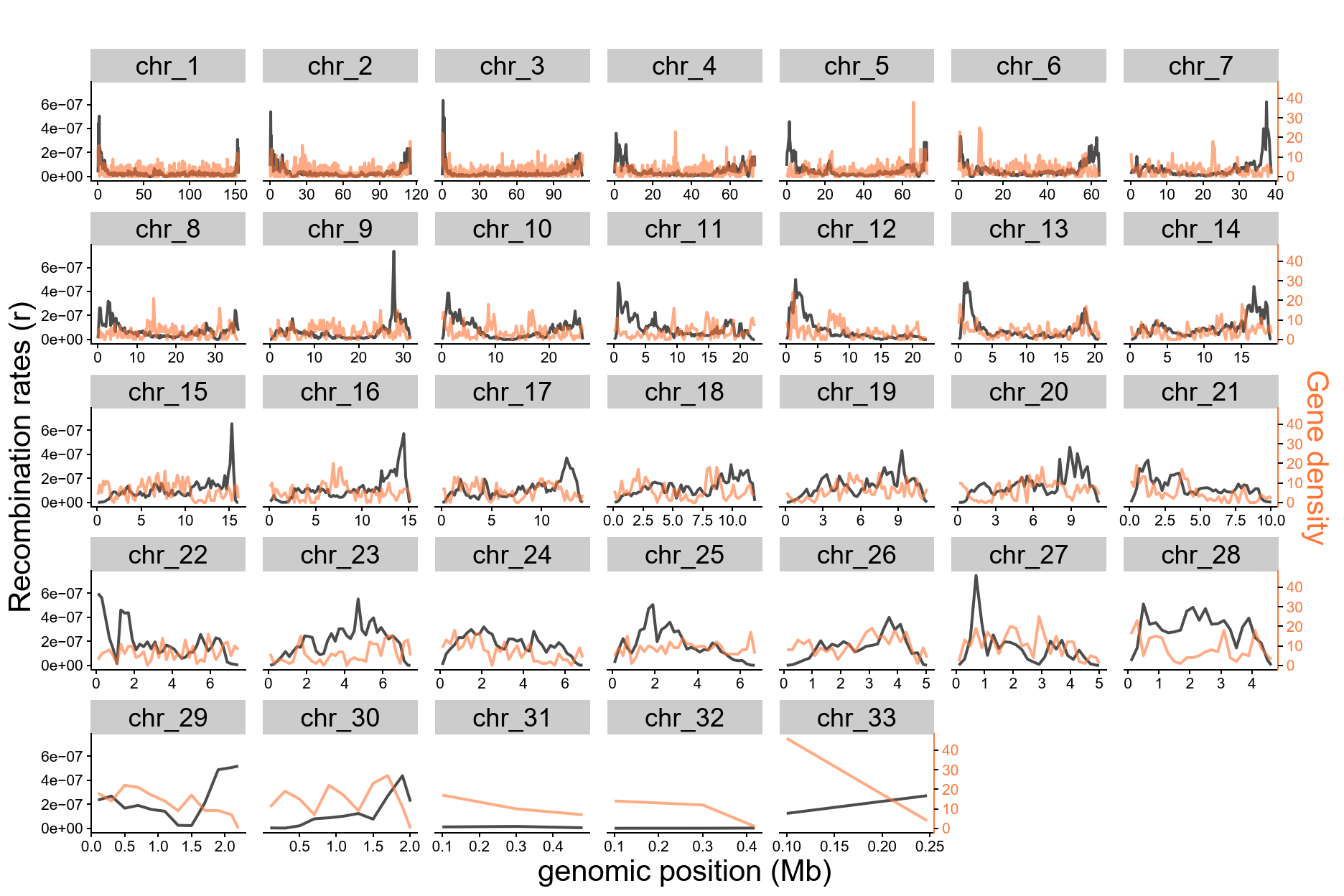


**Figure S5.** Complexity distribution calculated in 10 kb overlapping windows across all chromosomes.


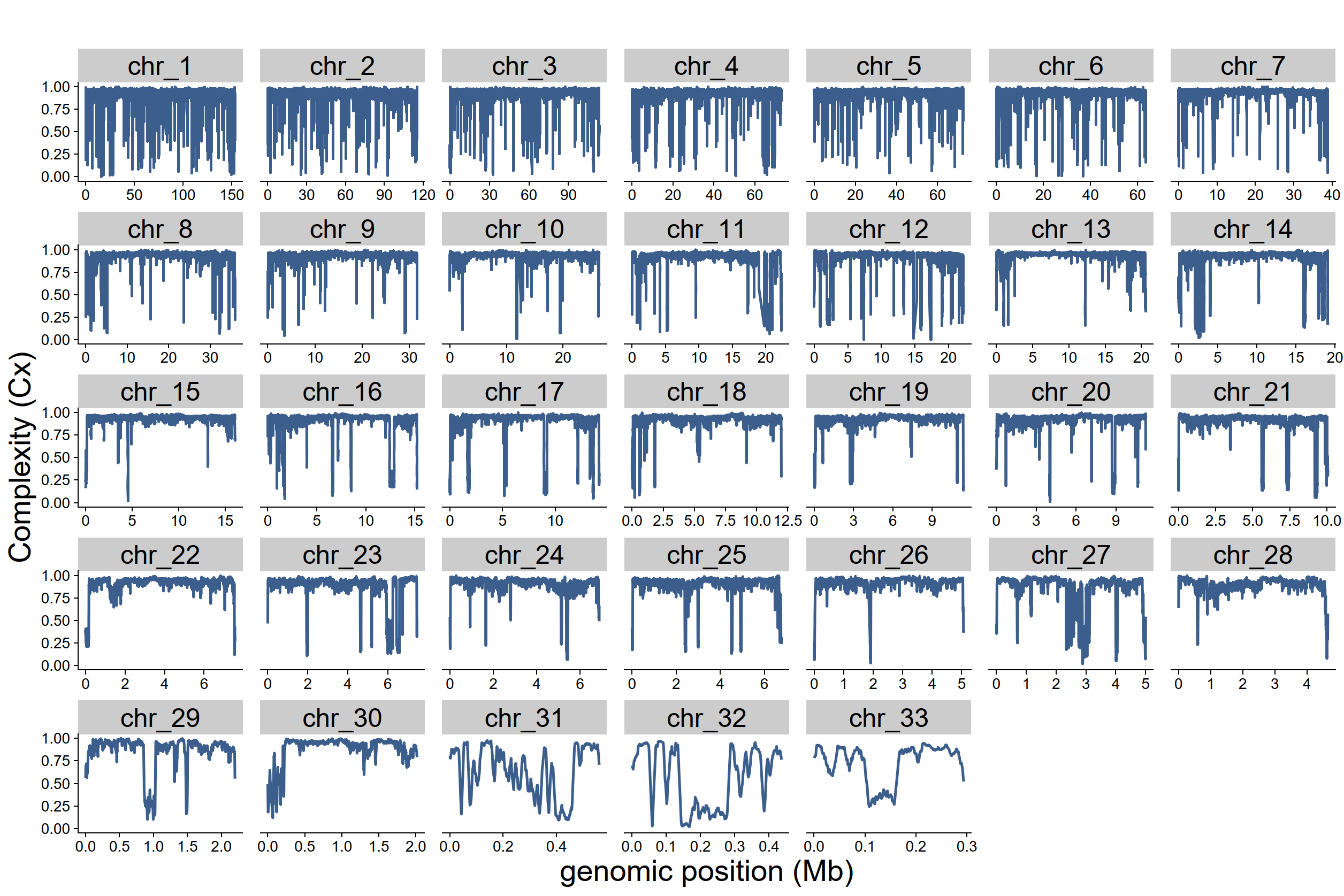


**Figure S6** Distribution of LTR retrotransposons coverage (right axis; purple) and recombination rates in 200 kb windows (black; left axis) across all chromosomes.


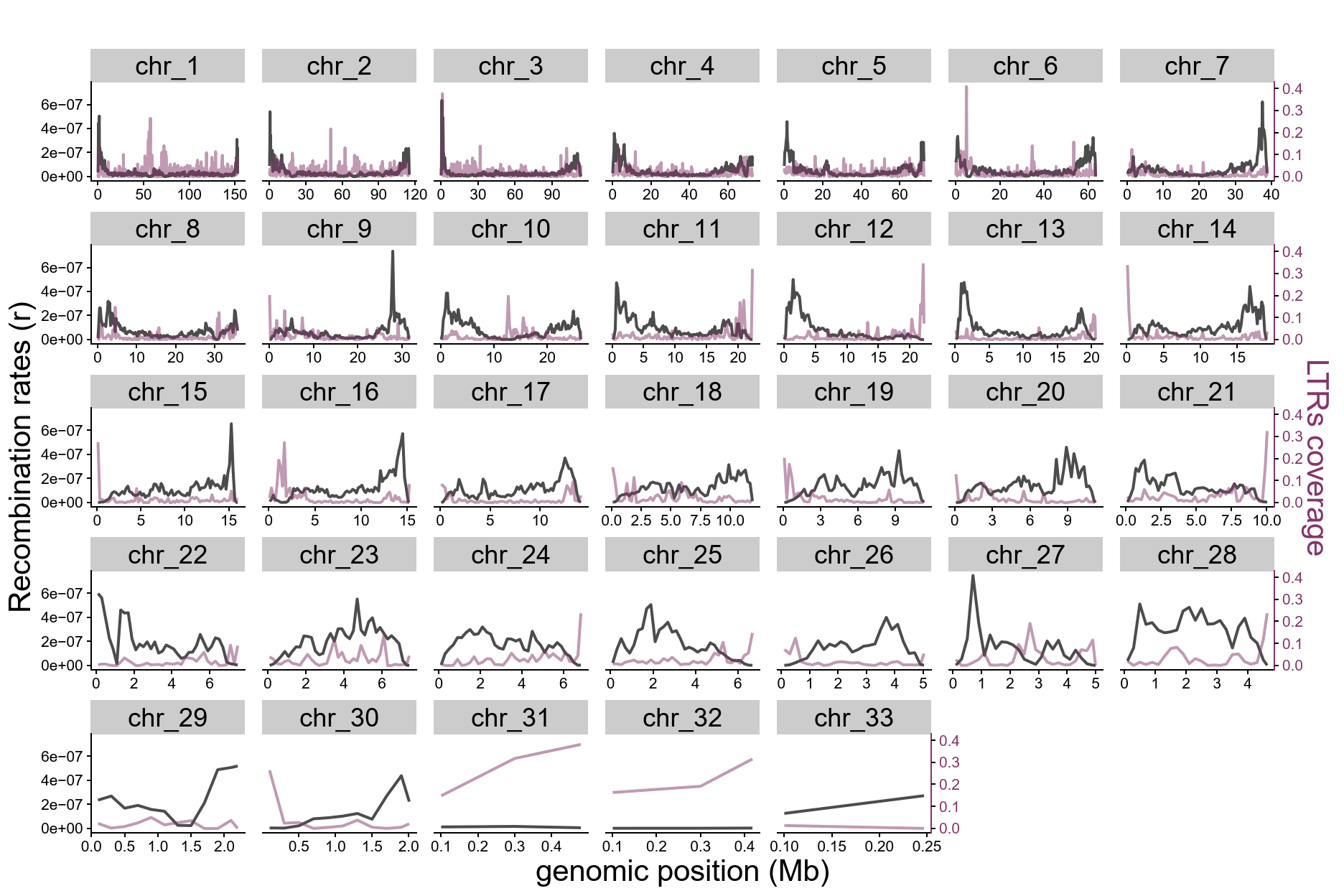


**Figure S7** Distribution of LINE retrotransposons coverage (right axis; green) and recombination rates in 200 kb windows (left axis; black) across all chromosomes.


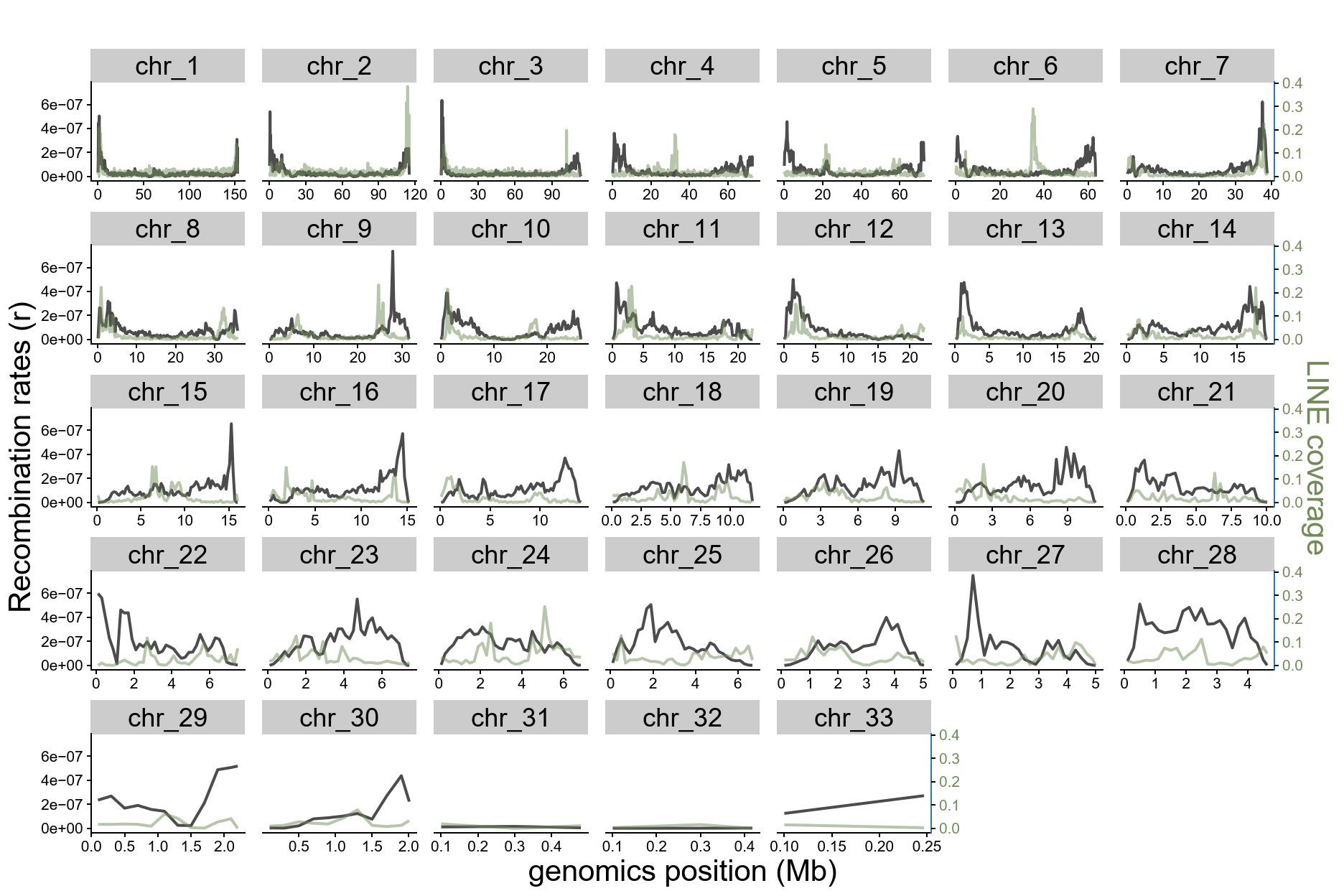


**Figure S8** Distribution of SINE retrotransposons coverage (blue; right axis) and recombination rates in 200 kb windows (black; left axis) across all chromosomes.


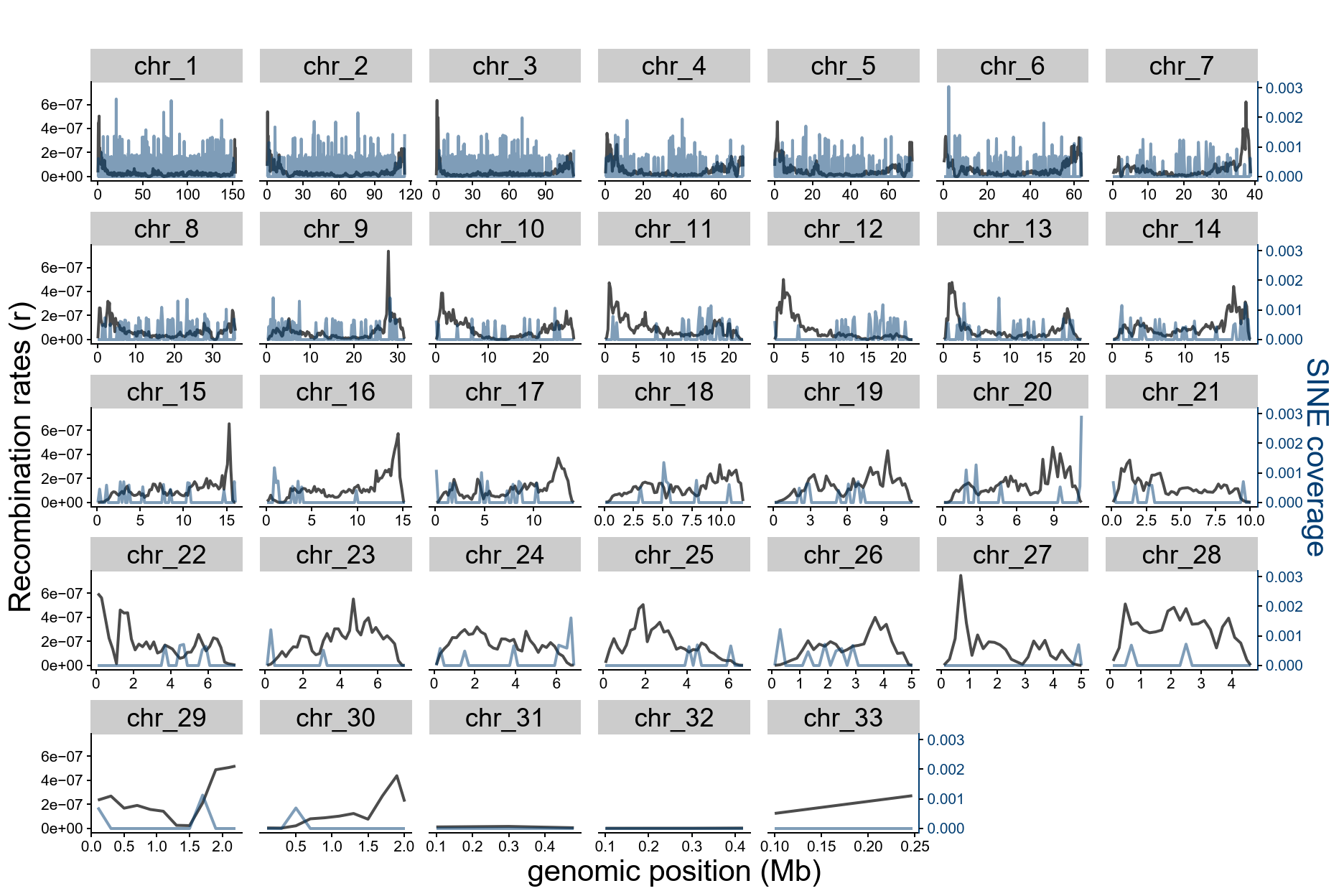


**Figure S9** Distribution of recombination rates using five samples for blackcaps (dark red) and garden warblers (cyan). Recombination rates were calculated in 50 kb non-overlapping windows across all chromosomes. Kendall non-parametric correlation coefficients reported for each chromosome.


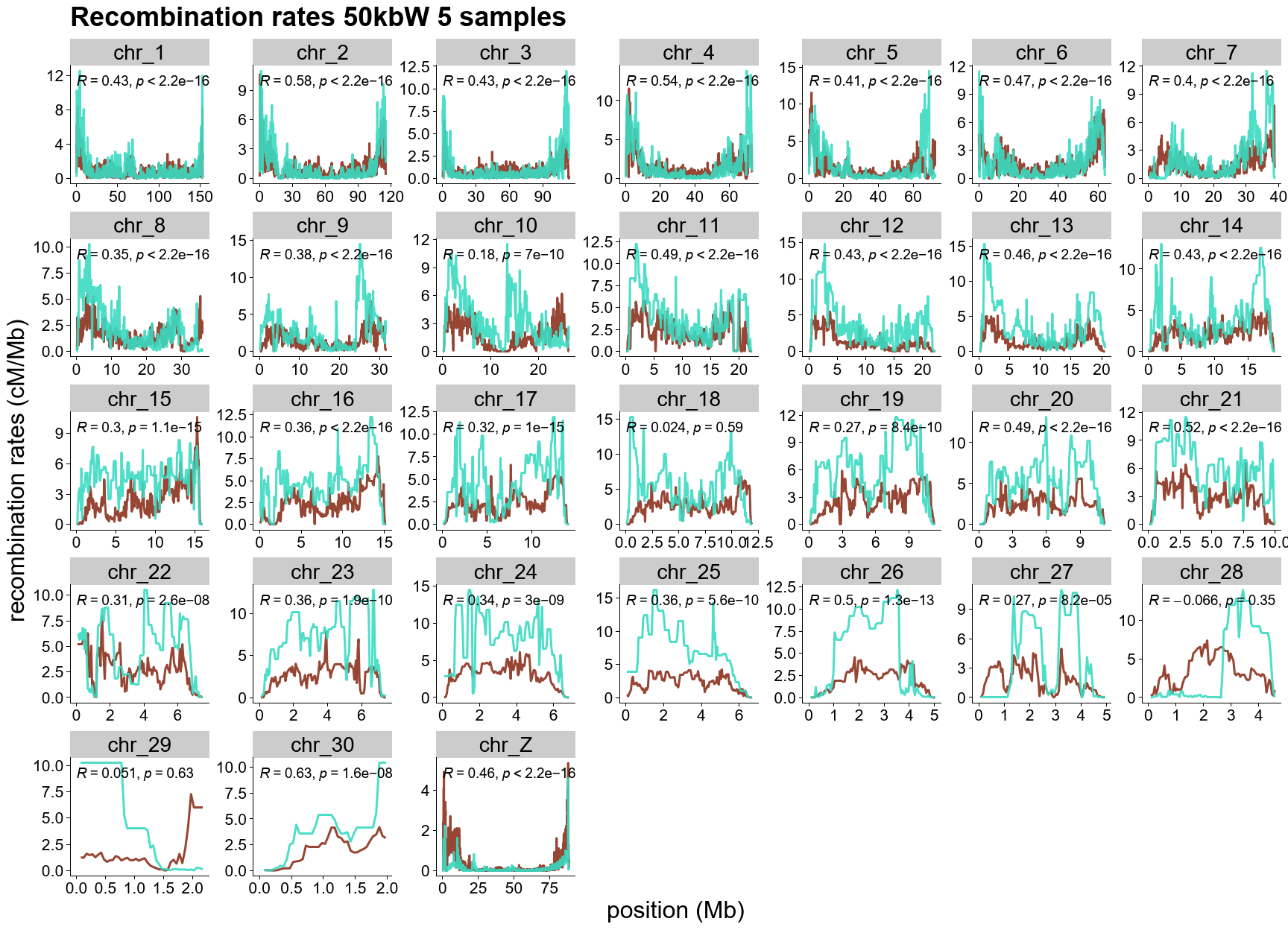


**References**

Akesson, M., Hansson, B., Hasselquist, D., & Bensch, S. (2007). Linkage mapping of AFLP markers in a wild population of great reed warblers: Importance of heterozygosity and number of genotyped individuals. *Molecular Ecology*, *16*(11), 2189–2202. https://doi.org/10.1111/j.1365-294X.2007.03290.x

Backström, N., Forstmeier, W., Schielzeth, H., Mellenius, H., Nam, K., Bolund, E., Webster, M. T., Öst, T., Schneider, M., Kempenaers, B., & Ellegren, H. (2010). The recombination landscape of the zebra finch *Taeniopygia guttata* genome. *Genome Research*, *20*(4), 485–495. <https://doi.org/10.1101/gr.101410.109>

Calderón, P. L., & Pigozzi, M. I. (2006). MLH1-focus mapping in birds shows equal recombination between sexes and diversity of crossover patterns. *Chromosome Research*, *14*(6), 605–612. <https://doi.org/10.1007/s10577-006-1059-0>

del Priore, L., & Pigozzi, M. I. (2017). Broad-scale recombination pattern in the primitive bird Rhea americana (Ratites, Palaeognathae). *PLoS ONE*, *12*(11), e0187549. https://doi.org/10.1371/journal.pone.0187549

del Priore, L., & Pigozzi, M. I. (2020). MLH1 focus mapping in the guinea fowl (*Numida meleagris)* give insights into the crossover landscapes in birds. *PLoS ONE*, *15*(10), e0240245. https://doi.org/10.1371/journal.pone.0240245

Groenen, M. A. M., Cheng, H. H., Bumstead, N., Benkel, B. F., Briles, W. E., Burke, T., Burt, D. W., Crittenden, L. B., Dodgson, J., Hillel, J., Lamont, S., de Leon, A. P., Soller, M., Takahashi, H., & Vignal, A. (2000). A Consensus Linkage Map of the Chicken Genome. *Genome Research*, *10*(1), 137–147.

Groenen, M. A. M., Wahlberg, P., Foglio, M., Cheng, H. H., Megens, H.-J., Crooijmans, R. P. M. A., Besnier, F., Lathrop, M., Muir, W. M., Wong, G. K.-S., Gut, I., & Andersson, L. (2009). A high-density SNP-based linkage map of the chicken genome reveals sequence features correlated with recombination rate. *Genome Research*, *19*(3), 510–519. https://doi.org/10.1101/gr.086538.108

Hansson, B., Akesson, M., Slate, J., & Pemberton, J. M. (2005). Linkage mapping reveals sex-dimorphic map distances in a passerine bird. *Proceedings. Biological Sciences*, *272*(1578), 2289–2298. <https://doi.org/10.1098/rspb.2005.3228>

Huang, Y., Zhao, Y., Haley, C. S., Hu, S., Hao, J., Wu, C., & Li, N. (2006). A Genetic and Cytogenetic Map for the Duck (*Anas platyrhynchos*). *Genetics*, *173*(1), 287–296. https://doi.org/10.1534/genetics.105.053256

Hagen, I. J., Lien, S., Billing, A. M., Elgvin, T. O., Trier, C., Niskanen, A. K., Tarka, M., Slate, J., Sætre, G.-P., & Jensen, H. (2020). A genome-wide linkage map for the house sparrow (*Passer domesticus*) provides insights into the evolutionary history of the avian genome. *Molecular Ecology Resources*, *20*(2), 544–559. <https://doi.org/10.1111/1755-0998.13134>

Kawakami T, Mugal CF, Suh A, Nater A, Burri R, Smeds L, Ellegren H. 2017. Whole-genome patterns of linkage disequilibrium across flycatcher populations clarify the causes and consequences of fine-scale recombination rate variation in birds. Molecular Ecology. 26(16):4158–4172. doi:10.1111/mec.14197.

Kayang, B. B., Vignal, A., Inoue-Murayama, M., Miwa, M., Monvoisin, J. L., Ito, S., & Minvielle, F. (2004). A first-generation microsatellite linkage map of the Japanese quail. *Animal Genetics*, *35*(3), 195–200. <https://doi.org/10.1111/j.1365-2052.2004.01135.x>

Kikuchi, S., Fujima, D., Sasazaki, S., Tsuji, S., Mizutani, M., Fujiwara, A., & Mannen, H. (2005). Construction of a genetic linkage map of Japanese quail (*Coturnix japonica*) based on AFLP and microsatellite markers. *Animal Genetics*, *36*(3), 227–231. <https://doi.org/10.1111/j.1365-2052.2005.01295.x>

Лисачев, А. П., Малиновская, Л. П., Друзяка, А. В., Бородин, П. М., & Торгашева, А. А. (2017). Синапсис и рекомбинация аутосом и половых хромосом у двух видов крачек (Sternidae, Charadriiformes, Aves). *Вавиловский журнал генетики и селекции*, *21*(2), 2. https://doi.org/10.18699/VJ17.245

Malinovskaya, L., Shnaider, E., Borodin, P., & Torgasheva, A. (2018). Karyotypes and recombination patterns of the Common Swift (*Apus apus* Linnaeus, 1758) and Eurasian Hobby (*Falco subbuteo* Linnaeus, 1758). *Avian Research*, *9*(1), 4. https://doi.org/10.1186/s40657-018-0096-7

Malinovskaya, L. P., Tishakova, K. V., Volkova, N. A., Torgasheva, A. A., Tsepilov, Y. A., & Borodin, P. M. (2019). Interbreed variation in meiotic recombination rate and distribution in the domestic chicken Gallus gallus. *Archives Animal Breeding*, *62*(2), 403–411. https://doi.org/10.5194/aab-62-403-2019

Peñalba JV, Deng Y, Fang Q, Joseph L, Moritz C, Cockburn A. 2020. Genome of an iconic Australian bird: High-quality assembly and linkage map of the superb fairy-wren (*Malurus cyaneus*). Molecular Ecology Resources. 20(2):560–578. doi:10.1111/1755-0998.13124.

Pigozzi, M. I., & del Priore, L. (2016). Meiotic recombination analysis in female ducks (*Anas platyrhynchos*). *Genetica*, *144*(3), 307–312. https://doi.org/10.1007/s10709-016-9899-9

Pigozzi, M. I., & Solari, A. J. (1999). Equal frequencies of recombination nodules in both sexes of the pigeon suggest a basic difference with eutherian mammals. *Genome*, *42*(2), 315–321. https://doi.org/10.1139/g98-137

Pigozzi, M. I. (2001). Distribution of MLH1 foci on the synaptonemal complexes of chicken oocytes. *Cytogenetic and Genome Research*, *95*(3–4), 129–133. <https://doi.org/10.1159/000059334>

Reed, K. M., Chaves, L. D., & Mendoza, K. M. (2007). An integrated and comparative genetic map of the turkey genome. *Cytogenetic and Genome Research*, *119*(1–2), 113–126. <https://doi.org/10.1159/000109627>

Robledo-Ruiz, D. A., Gan, H. M., Kaur, P., Dudchenko, O., Weisz, D., Khan, R., Lieberman Aiden, E., Osipova, E., Hiller, M., Morales, H. E., Magrath, M. J. L., Clarke, R. H., Sunnucks, P., & Pavlova, A. (2022). Chromosome-length genome assembly and linkage map of a critically endangered Australian bird: The helmeted honeyeater. *GigaScience*, *11*, giac025. <https://doi.org/10.1093/gigascience/giac025>

Singhal S, Leffler EM, Sannareddy K, Turner I, Venn O, Hooper DM, Strand AI, Li Q, Raney B, Balakrishnan CN, et al. 2015. Stable recombination hotspots in birds. Science. 350(6263):928–932. doi:10.1126/science.aad0843.

Stapley, J., Birkhead, T. R., Burke, T., & Slate, J. (2010). Pronounced inter- and intrachromosomal variation in linkage disequilibrium across the zebra finch genome. *Genome Research*, *20*(4), 496–502. <https://doi.org/10.1101/gr.102095.109>

Torgasheva, A. A., & Borodin, P. M. (2017). Immunocytological Analysis of Meiotic Recombination in the Gray Goose (*Anser anser*). *Cytogenetic and Genome Research*, *151*(1), 27–35. https://doi.org/10.1159/000458741

van Oers, K., Santure, A. W., De Cauwer, I., van Bers, N. E., Crooijmans, R. P., Sheldon, B. C., Visser, M. E., Slate, J., & Groenen, M. A. (2014). Replicated high-density genetic maps of two great tit populations reveal fine-scale genomic departures from sex-equal recombination rates. *Heredity*, *112*(3), 307–316. https://doi.org/10.1038/hdy.2013.107
